# Supplementary material for: Mapping the Neuroanatomy of Dystonia Using Causal Brain Lesions
Source: medRxiv. 2025 Dec 11:2025.12.07.25341373. Preprint. [Version 1] doi: 10.64898/2025.12.07.25341373 (PMC12709489; doi:10.64898/2025.12.07.25341373)
Supplement: Supplement 1 [file media-1.pdf]

Supplementary file 1. Systematic search syntax

Search of 'all fields' conducted in Pubmed including cases up to July 2022:

(dystonia OR blepharospasm OR meige OR meige's) AND (lesion OR lesions OR stroke OR infarct* OR ischem* OR ischaem* OR hemorrhag* OR haemorrhag* OR tumor OR tumour OR plaque*).

Search of titles and abstracts and keywords conducted in Embase including cases up to September 2022:

('dystonia':ti,ab,kw OR 'blepharospasm':ti,ab,kw OR 'meige':ti,ab,kw OR 'meige/s':ti,ab,kw) AND ('(lesion':ti,ab,kw OR 'lesions':ti,ab,kw OR 'stroke':ti,ab,kw OR 'infarct*':ti,ab,kw OR 'ischem*':ti,ab,kw OR 'ischaem*':ti,ab,kw OR 'hemorrhag*':ti,ab,kw OR 'haemorrhag*':ti,ab,kw OR 'plaque*':ti,ab,kw OR 'tumor':ti,ab,kw OR 'tumour)':ti,ab,kw).

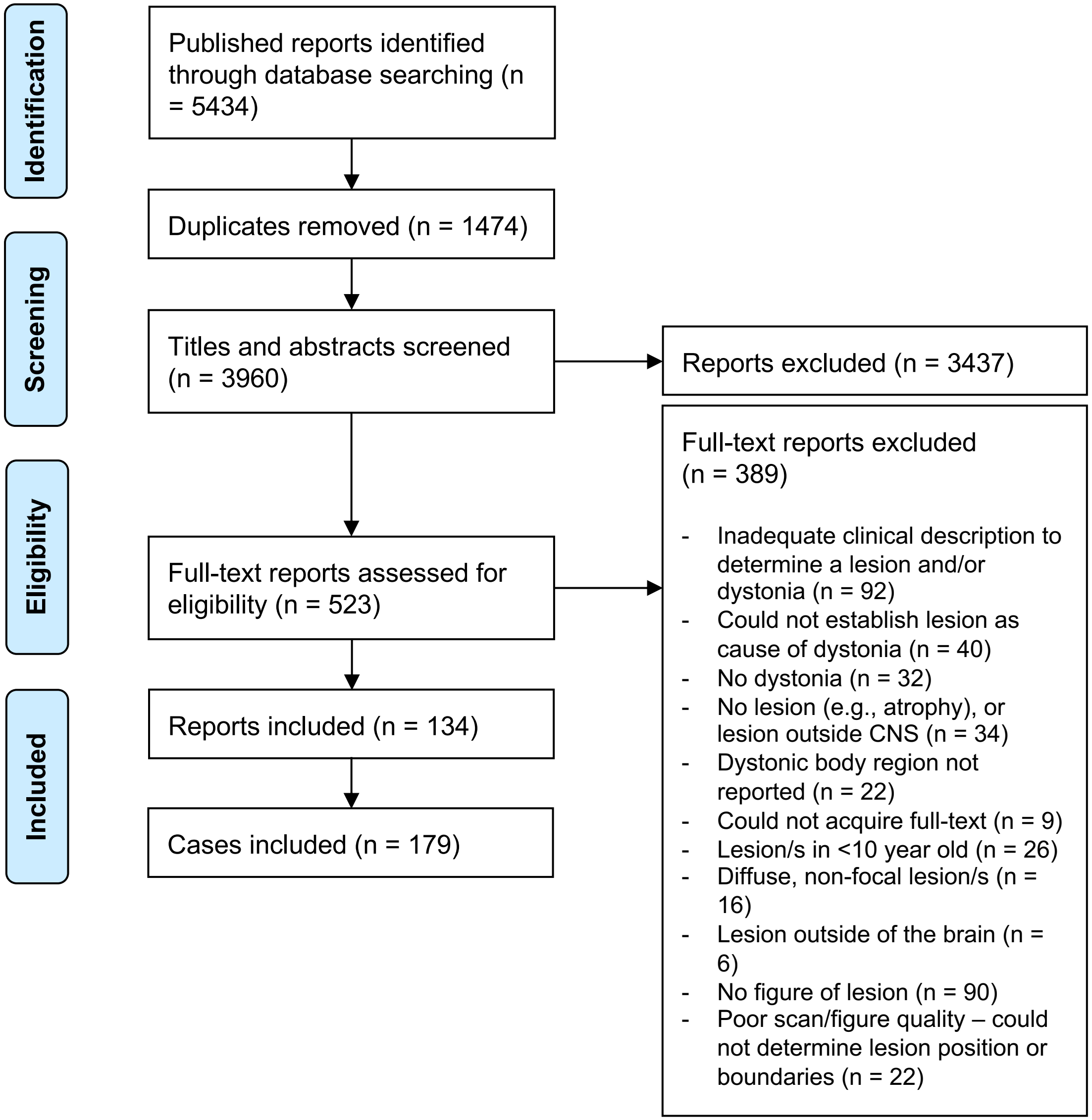

Supplementary File 2. Systematic search process. Flowchart adapted from Liberati et al. (2009). There were more cases than published reports because some reports contained multiple cases.

Supplementary File 3. Post-stroke non-specific neurological disorders dataset description

500 post-stroke patients were prospectively enrolled from the Turku University Hospital Division of Clinical Neurosciences as part of an on-going project at Turku University Hospital (Turku, Finland). The project was approved by the local ethics committee and hospital district, and conducted according to the principles of the Declaration of Helsinki. All cases were above the age of 18 years, with an acute focal stroke, willing to participate in the study, and gave written informed consent. Cases with diffuse, non-parenchymal or non-focal cerebrovascular events or no visible lesion in clinical brain imaging were excluded. Licensed clinicians specialising in neurology performed clinical evaluations in consultation with a consultant neurologist (J.J.). Cases without dystonia were included here as a post-stroke comparison group (n = 499).

Supplementary file 4. Clinical characteristics of the included cases.

Case	Study	StudyCase	Age range	Sex	BodyDistribution	Bleph.	Face	Cervical	Arm	Hand	Trunk	Leg	Foot	Variability
1	Aasfara (2012)		15-19	Male	Focal	No	No	No	Yes	Yes	No	No	No	Persistent
2	Aguirregomozcorta (2008)		35-39	Female	Hemidystonia	No	No	No	Yes	Yes	No	Yes	No	Paroxysmal
3	Akin (2014)		65-69	Female	Focal	No	Yes	No	No	No	No	No	No	Persistent
4	Alarcon (2001)	1	40-44	Female	Focal	No	No	No	Yes	Yes	No	No	No	Persistent
5	Alvarez (2014)	1	35-39	Male	Focal	No	No	No	Yes	Yes	No	No	No	Persistent
6	Apaydin (1998)	1	70-74	Female	Focal	No	No	No	Yes	Yes	No	No	No	Persistent
7	Apaydin (1998)	2	60-64	Male	Hemidystonia	No	No	No	Yes	Yes	No	Yes	No	Persistent
8	Aramideh (1996)		45-49	Female	Focal	Yes	No	No	No	No	No	No	No	Persistent
9	Azevedo (2001)		25-29	Female	Focal	No	No	No	Yes	Yes	No	No	No	Persistent
10	Baldacci (2010)		45-49	Male	Hemidystonia	No	No	No	Yes	Yes	No	Yes	Yes	Paroxysmal
11	Batla (2015)	1	55-59	Female	Focal	No	No	Yes	No	No	No	No	No	NR
12	Batla (2015)	2	30-34	Male	Focal	No	No	Yes	No	No	No	No	No	NR
13	Batla (2015)	5	55-59	Male	Multifocal	No	No	Yes	Yes	No	No	No	No	NR
14	Batla (2015)	7	25-29	Male	Focal	No	No	Yes	No	No	No	No	No	NR
15	Berkovic (1987)	3	45-49	Male	Generalized	No	Yes	Yes	No	No	Yes	Yes	Yes	Persistent
16	Bogdanova-Mihaylova (2017)		40-44	Female	Focal	No	No	No	Yes	Yes	No	No	No	Paroxysmal
17	Braga-Neto (2008)	1	25-29	Female	Hemidystonia	No	No	No	Yes	Yes	No	No	No	Persistent
18	Brett (1981)		10-14	Female	Hemidystonia	No	No	No	Yes	Yes	No	No	Yes	Persistent
19	Buerger (2014)		20-24	Male	Hemidystonia	No	Yes	No	Yes	No	No	No	No	Paroxysmal
20	Burguera (2001)	1	45-49	Male	Focal	No	No	No	No	Yes	No	No	No	Action_specific
21	Camac (1990)		50-54	Female	Hemidystonia	No	Yes	No	Yes	Yes	No	Yes	No	Paroxysmal
22	Chang (2002)	1	20-24	Male	Focal	No	No	Yes	No	No	No	No	No	Persistent
23	Chauhan (2009)	1	45-49	Female	Focal	Yes	No	No	No	No	No	No	No	Persistent
24	Cho (2000)	1	65-69	Female	Focal	No	No	No	No	Yes	No	No	No	Persistent
25	Choi (1993)	1	55-59	Male	Focal	No	No	No	No	Yes	No	No	No	Persistent
26	Choi (1993)	3	15-19	Male	Focal	No	No	No	No	No	No	No	Yes	Persistent
27	Choi (2015)		65-69	Male	Multifocal	No	Yes	No	Yes	Yes	No	Yes	Yes	Persistent
28	Comoglu (2002)	1	60-64	Male	Focal	Yes	No	No	No	No	No	No	No	Persistent
29	Day (1986)		75-79	Female	Segmental	Yes	Yes	No	No	No	No	No	No	Persistent
30	Defebvre (1995)	1	60-64	Female	Multifocal	No	No	No	Yes	Yes	No	Yes	Yes	Persistent
31	Deleu (2000)		15-19	Female	Focal	No	No	No	No	Yes	No	No	No	Persistent
32	Demierre (1983)	3	15-19	Male	Hemidystonia	No	No	No	Yes	Yes	No	Yes	Yes	Persistent

33	Di Capua (2001)		10-14	Female	Focal	No	No	No	No	Yes	No	No	No	Action_specific
34	Dietrichs (2000)		70-74	Female	Focal	No	Yes	No	No	No	No	No	No	Persistent
35	Dinkelbach (2015)		45-49	Female	Multifocal	No	No	Yes	No	No	Yes	No	No	Persistent
36	Esteban Munoz (1996)		45-49	Female	Focal	No	No	No	Yes	Yes	No	No	No	Persistent
37	Franzini (2009)		30-34	Female	Focal	No	No	No	No	No	No	No	Yes	Persistent
38	Fuller (2013)		40-44	Male	Generalized	No	No	Yes	Yes	Yes	Yes	Yes	No	Persistent
39	Galvez-Ruiz (2014)	1			Focal	Yes	No	No	No	No	No	No	No	NR
40	Ghika (1994)	1	90-94	Female	Focal	No	No	No	No	Yes	No	No	No	Persistent
41	Ghika (1994)	2	60-64	Male	Focal	No	No	No	No	Yes	No	No	No	Persistent
42	Ghika (1994)	3	30-34	Male	Focal	No	No	No	Yes	Yes	No	No	No	Persistent
43	Gilbert (2012)		30-34	Female	Focal	Yes	No	No	No	No	No	No	No	Persistent
44	Gille (1996)		65-69	Male	Hemidystonia	No	No	No	Yes	Yes	No	Yes	Yes	Persistent
45	Grandas (1989)		35-39	Male	Generalized	No	No	No	Yes	Yes	Yes	Yes	Yes	Persistent
46	Grandas (2004)		65-69	Female	Focal	Yes	No	No	No	No	No	No	No	Persistent
47	Guak (2002)		20-24	Female	Multifocal	No	Yes	No	Yes	No	No	No	No	Paroxysmal
48	Hamasaki (2008)	1	55-59	Male	Hemidystonia	No	No	No	Yes	Yes	No	Yes	No	Persistent
49	Hamasaki (2008)	2	70-74	Female	Hemidystonia	Yes	Yes	No	Yes	No	No	Yes	No	Persistent
50	Hawker (1990)	1	55-59	Female	Multifocal	No	No	No	Yes	Yes	No	Yes	Yes	Persistent
51	Hawker (1990)	3	45-49	Male	Focal	No	No	No	No	No	No	No	Yes	Persistent
52	Hsieh (2014)		70-74	Male	Focal	Yes	No	No	No	No	No	No	No	Persistent
53	Isaac (1989)		25-29	Male	Focal	No	No	Yes	No	No	No	No	No	Persistent
54	Jacob (1995)		40-44	Male	Segmental	Yes	Yes	No	No	No	No	No	No	Persistent
55	Jankovic (1983)	1	55-59	Female	Focal	Yes	No	No	No	No	No	No	No	Persistent
56	Jankovic (1983)	4	50-54	Female	Segmental	Yes	Yes	No	No	No	No	No	No	Persistent
57	Jankovic (1986)		50-54	Male	Focal	Yes	No	No	No	No	No	No	No	Persistent
58	Jung (2016)		45-49	Female	Hemidystonia	No	No	No	No	No	No	Yes	Yes	Paroxysmal
59	Kajimoto (2004)	1	80-84	Female	Focal	No	No	Yes	No	No	No	No	No	Persistent
60	Karsidag (1998)	1	65-69	Female	Focal	No	No	No	No	Yes	No	No	No	Action_specific
61	Karsidag (1998)	2	60-64	Female	Focal	No	No	No	No	Yes	No	No	No	Persistent
62	Karsidag (1998)	4	45-49	Male	Focal	No	No	No	No	Yes	No	No	No	Persistent
63	Karsidag (1998)	5	60-64	Male	Focal	No	No	No	No	Yes	No	No	No	Persistent
64	Karsidag (1998)	7	50-54	Female	Generalized	No	No	Yes	No	Yes	Yes	Yes	No	Persistent
65	Karsidag (1998)	8	65-69	Female	Focal	No	No	No	No	Yes	No	No	No	Persistent
66	Karsidag (1998)	9	50-54	Female	Focal	No	No	No	No	Yes	No	No	No	Persistent

67	Keane (1985)		30-34	Male	Multifocal	Yes	No	No	No	Yes	No	No	No	Persistent
68	Kemp (2012)		40-44	Male	Focal	No	No	No	No	No	No	Yes	No	Action_specific
69	Kim (2009)		70-74	Male	Focal	No	Yes	No	No	No	No	No	No	Persistent
70	Kirton (2001)		60-64	Female	Segmental	Yes	Yes	Yes	No	No	No	No	No	Paroxysmal
71	Koc (2010)		10-14	Male	Hemidystonia	No	No	No	Yes	No	No	No	No	Persistent
72	Koch (2006)		60-64	Male	Focal	No	No	No	No	No	No	Yes	Yes	Paroxysmal
73	Krauss, Mohadjer, Wakhloo (1991)		35-39	Female	Multifocal	Yes	Yes	Yes	No	No	No	Yes	No	Persistent
74	Krystkowiak (1998)	5	55-59		Focal	No	No	No	Yes	Yes	No	No	No	NR
75	Kulisevsky (1993)		55-59	Female	Focal	Yes	No	No	No	No	No	No	No	Paroxysmal
76	Kuoppamaki (2002)	2	20-24	Female	Generalized	No	Yes	No	Yes	Yes	Yes	Yes	Yes	Persistent
77	Lambrecq (2010)		20-24	Male	Multifocal	Yes	No	Yes	No	No	No	No	No	Persistent
78	Larumbe (1993)		10-14	Female	Generalized	Yes	No	No	Yes	No	Yes	Yes	No	Action_specific
79	Lauterbach (1994)		60-64	Female	Focal	Yes	No	No	No	No	No	No	No	Persistent
80	LeDoux (2003)	1	55-59	Male	Focal	No	No	Yes	No	No	No	No	No	Persistent
81	LeDoux (2003)	3	65-69	Female	Focal	No	No	Yes	No	No	No	No	No	Persistent
82	LeDoux (2003)	4	70-74	Male	Focal	No	No	Yes	No	No	No	No	No	Persistent
83	Lee, Lee (1996)		30-34	Male	Multifocal	No	Yes	No	No	Yes	No	No	No	Persistent
84	Lee, Rinne (1994)	1	10-14	Female	Hemidystonia	No	No	No	Yes	Yes	No	Yes	No	Persistent
85	Leenders (1986)		40-44	Male	Multifocal	Yes	No	No	Yes	Yes	No	Yes	No	Persistent
86	Lehericy (1996)	3	55-59	Female	Multifocal	No	No	No	No	Yes	No	Yes	Yes	Persistent
87	Lehericy (1996)	5	70-74	Male	Focal	No	No	No	Yes	Yes	No	No	No	Persistent
88	Lehericy (2001)	1	60-64		Focal	No	No	No	No	Yes	No	No	No	Persistent
89	Lehericy (2001)	5	45-49		Focal	No	No	No	No	Yes	No	No	No	Persistent
90	Lehericy (2001)	6	65-69		Focal	No	No	No	No	Yes	No	No	No	Persistent
91	Lehericy (2001)	7	50-54		Focal	No	No	No	No	Yes	No	No	No	Persistent
92	Lera (2000)	2	75-79	Female	Focal	No	No	No	Yes	Yes	No	No	No	Persistent
93	Lera (2000)	7	70-74	Female	Focal	No	No	No	Yes	No	No	No	No	Persistent
94	Liepert (2003)	1	55-59	Male	Focal	No	No	No	No	Yes	No	No	No	Action_specific
95	Loher (2009)	1	30-34	Male	Multifocal	No	Yes	Yes	Yes	Yes	No	Yes	No	Persistent
96	Loher (2009)	2	40-44	Male	Hemidystonia	No	No	Yes	Yes	No	No	Yes	No	Persistent
97	Loher (2009)	4	55-59	Male	Hemidystonia	Yes	No	Yes	Yes	No	No	No	No	Persistent
98	Mahant (2003)		35-39	Female	Focal	No	No	No	No	No	No	Yes	Yes	Persistent
99	Marsden (1985)	8	10-14	Male	Hemidystonia	No	No	No	Yes	Yes	No	Yes	No	Persistent
100	Marsden (1985)	10	75-79	Female	Focal	No	No	No	No	Yes	No	No	No	Persistent

101	Marsden (1985)	11	20-24	Female	Hemidystonia	No	No	No	Yes	Yes	No	No	Yes	Persistent
102	Marsden (1985)	22	60-64	Female	Hemidystonia	No	No	No	Yes	Yes	No	Yes	No	Persistent
103	Micheli (1998)		10-14	Female	Hemidystonia	No	No	No	Yes	Yes	No	No	Yes	Persistent
104	Miletic (2015)		70-74	Female	Hemidystonia	No	No	No	Yes	No	No	Yes	Yes	Persistent
105	Miranda (1998)		50-54	Male	Focal	Yes	No	No	No	No	No	No	No	Persistent
106	Molho (1993)	1	65-69	Female	Focal	No	No	Yes	No	No	No	No	No	Persistent
107	Molho (1993)	2	40-44	Female	Focal	No	No	Yes	No	No	No	No	No	Persistent
108	Munchau (2000)	1	25-29	Male	Hemidystonia	No	No	No	Yes	Yes	No	Yes	Yes	Persistent
109	Munchau (2000)	3	25-29	Male	Hemidystonia	No	No	Yes	Yes	Yes	No	No	Yes	Persistent
110	Munchau (2000)	4	25-29	Male	Focal	No	No	No	No	No	No	Yes	Yes	Persistent
111	Nishimura (2014)		60-64	Male	Hemidystonia	No	No	No	Yes	Yes	No	No	Yes	Persistent
112	Nociti (2011)	1	30-34	Female	Focal	Yes	No	No	No	No	No	No	No	Persistent
113	Nociti (2011)	2	45-49	Male	Focal	Yes	No	No	No	No	No	No	No	Persistent
114	O'Rourke (2006)		35-39	Female	Multifocal	Yes	No	Yes	No	No	No	No	No	Paroxysmal
115	Olgiati (2016)	1	15-19	Male	Generalized	No	No	Yes	Yes	Yes	Yes	Yes	Yes	Persistent
116	Panda (2014)		10-14	Female	Focal	No	No	No	No	Yes	No	No	No	Persistent
117	Pandey (2014)		15-19	Female	Hemidystonia	No	No	No	No	Yes	No	No	Yes	Persistent
118	Persing (1990)		55-59	Female	Segmental	Yes	Yes	No	No	No	No	No	No	Persistent
119	Pettigrew (1985)	16	60-64	Male	Hemidystonia	No	No	No	No	Yes	No	No	Yes	NR
120	Plant (1989)		30-34	Female	Focal	No	No	Yes	No	No	No	No	No	Persistent
121	Powers (1985)		60-64	Male	Multifocal	Yes	No	No	No	Yes	No	No	No	Persistent
122	Rana (2014)		65-69	Female	Hemidystonia	No	No	No	Yes	Yes	No	No	Yes	Persistent
123	Riley (1996)		65-69	Male	Focal	No	Yes	No	No	No	No	No	No	Paroxysmal
124	Sabrie (2015)		25-29	Male	Focal	No	No	No	Yes	Yes	No	No	No	Paroxysmal
125	Salerno (1993)		40-44	Female	Generalized	No	Yes	Yes	Yes	No	Yes	No	No	Persistent
126	Schulze-Bonhage (1995)		40-44	Male	Focal	No	No	Yes	No	No	No	No	No	Persistent
127	Schwartz (1995)		60-64	Male	Focal	No	Yes	Yes	No	No	No	No	No	Persistent
128	Seet (2005)		65-69	Male	Segmental	No	No	Yes	Yes	No	No	No	No	Paroxysmal
129	Shen (2016)		20-24	Female	Focal	No	No	No	No	Yes	No	No	No	Persistent
130	Singer (1998)		65-69	Female	Focal	Yes	No	No	No	No	No	No	No	Persistent
131	Takahashi (2009)		40-44	Female	Focal	No	No	No	No	Yes	No	No	No	Persistent
132	Tamburin (2005)	2	35-39	Female	Multifocal	No	No	No	Yes	Yes	No	Yes	Yes	Paroxysmal
133	Tan (2005)		40-44	Male	Hemidystonia	No	No	No	Yes	Yes	No	Yes	Yes	Diurnal
134	Tranchant (1991)		50-54	Female	Focal	No	No	Yes	No	No	No	No	No	NR

135	Trankle (1997)		25-29	Male	Focal	No	No	No	No	Yes	No	No	No	NR
136	Trompetto (2012)	1	50-54		Hemidystonia	No	No	No	Yes	No	No	No	Yes	Persistent
137	Trompetto (2012)	3	60-64		Hemidystonia	No	No	No	Yes	No	No	No	Yes	Persistent
138	Trompetto (2012)	6	45-49		Hemidystonia	No	No	No	Yes	Yes	No	No	Yes	Persistent
139	Trompetto (2012)	7	70-74		Focal	No	No	No	No	Yes	No	No	No	Persistent
140	Usmani (2011)	1	35-39	Male	Focal	No	No	Yes	No	No	No	No	No	Paroxysmal
141	Verghese (1999)		60-64	Female	Focal	Yes	No	No	No	No	No	No	No	Action_specific
142	Vidailhet (1999)	1	50-54	Male	Hemidystonia	No	Yes	No	Yes	Yes	No	No	No	Persistent
143	Vidailhet (1999)	3	50-54	Female	Focal	No	No	No	No	Yes	No	No	No	Persistent
144	Vidailhet (1999)	4	55-59	Male	Hemidystonia	No	No	No	Yes	Yes	No	No	Yes	Action_specific
145	Vidailhet (1999)	5	60-64	Male	Focal	No	No	No	No	Yes	No	No	No	Persistent
146	Vidailhet (1999)	7	55-59	Male	Focal	No	No	No	No	Yes	No	No	No	Persistent
147	Wali (2001)		25-29	Female	Focal	Yes	No	No	No	No	No	No	No	Persistent
148	Walker (2015)		55-59	Male	Generalized	No	No	Yes	Yes	No	Yes	No	No	Persistent
149	Wolz (2008)		40-44	Male	Generalized	No	Yes	Yes	No	No	Yes	No	No	Persistent
150	Wu (1992)		45-49	Female	Multifocal	No	No	Yes	Yes	Yes	No	No	Yes	Persistent
151	Zadro (2008)	1	45-49	Female	Focal	No	No	Yes	No	No	No	No	No	Persistent
152	Garbin Di Luca (2019)		50-54	Male	Multifocal	No	No	Yes	Yes	Yes	No	Yes	Yes	Paroxysmal
153	Pandey (2018)	7	55-59	Male	Focal	No	Yes	No	No	No	No	No	No	Persistent
154	Pandey (2018)	9	60-64	Male	Focal	No	Yes	No	No	No	No	No	No	Persistent
155	Pandey (2018)	11	65-69	Female	Focal	No	Yes	No	No	No	No	No	No	Persistent
156	Shin (2019)		55-59	Female	Focal	No	No	No	No	No	No	Yes	Yes	Persistent
157	Szejko (2020)		35-39	Male	Multifocal	No	No	No	No	Yes	No	No	Yes	Persistent
158	Hamed (2020)	6	60-64	Female	Focal	No	Yes	No	No	No	No	No	No	Persistent
159	Li (2019)	2	15-19	Male	Hemidystonia	No	No	No	Yes	Yes	No	No	Yes	Persistent
160	Li (2019)	3	25-29	Female	Generalized	No	Yes	Yes	Yes	Yes	Yes	Yes	Yes	Persistent
161	Li (2019)	5	35-39	Male	Focal	No	No	No	No	Yes	No	No	No	Persistent
162	Sul (2019)		40-44	Male	Focal	No	No	No	No	Yes	No	No	No	Persistent
163	Ghosh (2020)		25-29	Female	Focal	No	No	No	Yes	Yes	No	No	No	Persistent
164	Guimarães Rocha (2021)	1	50-54	Female	Hemidystonia	No	Yes	Yes	Yes	Yes	No	Yes	Yes	Persistent
165	Guimarães Rocha (2021)	2	10-14	Female	Hemidystonia	No	Yes	No	Yes	Yes	No	Yes	Yes	Persistent
166	Han (2020)		40-44	Male	Multifocal	No	Yes	No	No	Yes	No	No	No	Persistent
167	Meloni (2021)		40-44	Male	Hemidystonia	No	No	No	Yes	Yes	No	Yes	Yes	Persistent
168	Owen (2022)		35-39		Generalized	Yes	No	Yes	Yes	Yes	Yes	Yes	No	Persistent

169	Umeh (2012)		65-69	Male	Hemidystonia	No	No	No	Yes	Yes	No	No	Yes	Persistent
170	Wu, Zheng (2020)		15-19	Female	Generalized	No	Yes	Yes	Yes	Yes	Yes	Yes	Yes	NR
171	Wu, Su (2020)		15-19	Female	Focal	No	No	No	Yes	Yes	No	No	No	Persistent
172	Xu (2020)		70-74	Male	Focal	No	No	No	No	Yes	No	No	No	NR
173	Yu (2021)		20-24	Female	Multifocal	No	No	No	Yes	Yes	No	Yes	Yes	Persistent
174	Aldosary (2021)	1	10-14	Male	Focal	No	No	No	Yes	Yes	No	No	No	Action_specific
175	Smith (2021)		35-39	Male	Focal	No	Yes	No	No	No	No	No	No	Persistent
176	Funabe (2014)		60-64	Male	Focal	No	No	Yes	No	No	No	No	No	Persistent
177	Kim, Lee (2007)		35-39	Male	Focal	No	No	Yes	No	No	No	No	No	Persistent
178	Walker (2007)		40-44	Male	Focal	No	No	Yes	No	No	No	No	No	Persistent
179	Trompetto (2012)	9	80-84		Hemidystonia	No	No	No	Yes	Yes	No	No	Yes	Persistent

Case	Study	LatencyWeeks	DystonicTremor	AssociatedTremor	SensoryTrick
1	Aasfara (2012)	2	No	No	No
2	Aguirregomozcorta (2008)		No	No	No
3	Akin (2014)	.01	No	No	Yes
4	Alarcon (2001)	.5	Yes	No	
5	Alvarez (2014)	17	Yes	Yes	
6	Apaydin (1998)	13	Yes	No	
7	Apaydin (1998)	9	No	No	
8	Aramideh (1996)		No	No	
9	Azevedo (2001)		No	No	
10	Baldacci (2010)		No	No	No
11	Batla (2015)		Yes	No	Yes
12	Batla (2015)		No	No	
13	Batla (2015)		Yes	No	
14	Batla (2015)		Yes	No	
15	Berkovic (1987)		No	No	No
16	Bogdanova-Mihaylova (2017)	13	No	No	No
17	Braga-Neto (2008)		No	No	
18	Brett (1981)	1	No	No	
19	Buerger (2014)	3	No	No	
20	Burguera (2001)	1	No	No	
21	Camac (1990)		No	No	Yes
22	Chang (2002)	156	No	No	Yes
23	Chauhan (2009)		No	No	
24	Cho (2000)	.01	Yes	No	
25	Choi (1993)	52	No	No	
26	Choi (1993)	4	No	No	
27	Choi (2015)		No	No	
28	Comoglu (2002)	.01	No	No	
29	Day (1986)		No	Yes	
30	Defebvre (1995)	3	No	No	
31	Deleu (2000)		No	No	No
32	Demierre (1983)	4	No	No	

33	Di Capua (2001)		No	No	No
34	Dietrichs (2000)		Yes	No	Yes
35	Dinkelbach (2015)		No	No	Yes
36	Esteban Munoz (1996)	.01	Yes	No	
37	Franzini (2009)		No	No	No
38	Fuller (2013)		No	No	No
39	Galvez-Ruiz (2014)		NR	NR	
40	Ghika (1994)		Yes	Yes	Yes
41	Ghika (1994)		No	No	
42	Ghika (1994)		Yes	Yes	
43	Gilbert (2012)		No	No	
44	Gille (1996)	34	No	No	
45	Grandas (1989)		Yes	No	No
46	Grandas (2004)	.01	No	No	
47	Guak (2002)		No	No	
48	Hamasaki (2008)	13	No	No	
49	Hamasaki (2008)	14	No	No	
50	Hawker (1990)		No	No	
51	Hawker (1990)		Yes	Yes	
52	Hsieh (2014)		No	No	
53	Isaac (1989)	208	No	No	No
54	Jacob (1995)		No	No	
55	Jankovic (1983)	52	No	No	
56	Jankovic (1983)	156	No	No	
57	Jankovic (1986)	1.5	No	No	
58	Jung (2016)	21	No	No	No
59	Kajimoto (2004)	1.5	No	No	
60	Karsidag (1998)	39	No	No	
61	Karsidag (1998)	104	No	No	
62	Karsidag (1998)	156	Yes	No	
63	Karsidag (1998)	130	No	No	
64	Karsidag (1998)	4	No	No	
65	Karsidag (1998)	43	No	No	
66	Karsidag (1998)	4	Yes	No	

67	Keane (1985)	26	Yes	No	
68	Kemp (2012)	39	No	No	
69	Kim (2009)		No	No	
70	Kirton (2001)	104	No	No	
71	Koc (2010)	156	Yes	Yes	No
72	Koch (2006)		No	No	No
73	Krauss, Mohadjer, Wakhloo (1991)	5	No	No	
74	Krystkowiak (1998)	30	No	No	
75	Kulisevsky (1993)	13	No	No	
76	Kuoppamaki (2002)	8	No	No	
77	Lambrecq (2010)		No	No	
78	Larumbe (1993)	.5	No	No	
79	Lauterbach (1994)	.01	No	No	
80	LeDoux (2003)	.1	No	No	
81	LeDoux (2003)	.5	No	Yes	
82	LeDoux (2003)	.1	No	No	
83	Lee, Lee (1996)	4	Yes	No	
84	Lee, Rinne (1994)	52	No	No	
85	Leenders (1986)		No	No	
86	Lehericy (1996)	3	No	No	
87	Lehericy (1996)	4	No	No	
88	Lehericy (2001)		No	No	
89	Lehericy (2001)		No	No	
90	Lehericy (2001)		No	No	
91	Lehericy (2001)		Yes	No	
92	Lera (2000)	52	No	No	
93	Lera (2000)	.5	No	No	
94	Liepert (2003)		No	No	
95	Loher (2009)	13	Yes	No	
96	Loher (2009)	17	No	No	
97	Loher (2009)	4	Yes	Yes	
98	Mahant (2003)		No	No	No
99	Marsden (1985)	104	No	No	
100	Marsden (1985)	8	No	No	

101	Marsden (1985)	8	Yes	No	
102	Marsden (1985)	156	No	No	
103	Micheli (1998)	3	Yes	No	No
104	Miletic (2015)	.1	No	No	
105	Miranda (1998)	56	No	No	
106	Molho (1993)		Yes	No	Yes
107	Molho (1993)		No	No	Yes
108	Munchau (2000)	156	No	No	
109	Munchau (2000)		No	No	
110	Munchau (2000)		No	No	
111	Nishimura (2014)	1	No	No	
112	Nociti (2011)		No	No	
113	Nociti (2011)		No	No	
114	O'Rourke (2006)	.5	No	No	
115	Olgiati (2016)	.4	No	No	No
116	Panda (2014)	.01	Yes	No	
117	Pandey (2014)		No	No	
118	Persing (1990)		No	No	
119	Pettigrew (1985)	26	No	No	
120	Plant (1989)	52	No	No	Yes
121	Powers (1985)		No	No	
122	Rana (2014)		No	No	No
123	Riley (1996)		No	No	
124	Sabrie (2015)	.1	No	No	
125	Salerno (1993)	13	No	No	No
126	Schulze-Bonhage (1995)		No	No	
127	Schwartz (1995)		No	No	
128	Seet (2005)		No	Yes	No
129	Shen (2016)	2	No	No	
130	Singer (1998)		No	Yes	
131	Takahashi (2009)	52	No	No	No
132	Tamburin (2005)		No	No	
133	Tan (2005)	.01	No	No	
134	Tranchant (1991)		NR	NR	

135	Trankle (1997)	17	NR	NR	
136	Trompetto (2012)	20	No	No	
137	Trompetto (2012)	12	No	No	
138	Trompetto (2012)	24	Yes	No	
139	Trompetto (2012)	104	Yes	No	
140	Usmani (2011)	65	No	No	
141	Verghese (1999)		No	No	
142	Vidailhet (1999)	9	No	No	
143	Vidailhet (1999)	.01	No	No	
144	Vidailhet (1999)	.01	No	No	
145	Vidailhet (1999)	22	Yes	Yes	
146	Vidailhet (1999)	7	No	Yes	
147	Wali (2001)	4	No	No	
148	Walker (2015)	17	No	No	No
149	Wolz (2008)	.3	No	No	No
150	Wu (1992)	8	Yes	No	No
151	Zadro (2008)	.2	No	No	
152	Garbin Di Luca (2019)	.5	No	No	No
153	Pandey (2018)	.4	No	No	No
154	Pandey (2018)	20	Yes	No	No
155	Pandey (2018)	16	Yes	No	No
156	Shin (2019)	260	No	No	No
157	Szejko (2020)	.01	No	No	No
158	Hamed (2020)		No	No	No
159	Li (2019)	26	No	No	No
160	Li (2019)	52	No	No	No
161	Li (2019)	78	Yes	No	No
162	Sul (2019)	12	No	No	No
163	Ghosh (2020)		No	No	NR
164	Guimarães Rocha (2021)	26	Yes	No	NR
165	Guimarães Rocha (2021)		No	No	NR
166	Han (2020)		No	No	NR
167	Meloni (2021)	52	Yes	No	NR
168	Owen (2022)	104	No	No	NR

169	Umeh (2012)	13	No	No	NR
170	Wu, Zheng (2020)		NR	NR	NR
171	Wu, Su (2020)		Yes	No	NR
172	Xu (2020)		No	No	NR
173	Yu (2021)		Yes	No	NR
174	Aldosary (2021)		No	No	NR
175	Smith (2021)		No	No	NR
176	Funabe (2014)		No	No	NR
177	Kim, Lee (2007)		Yes	No	Yes
178	Walker (2007)	21	Yes	Yes	NR
179	Trompetto (2012)	28	Yes	No	

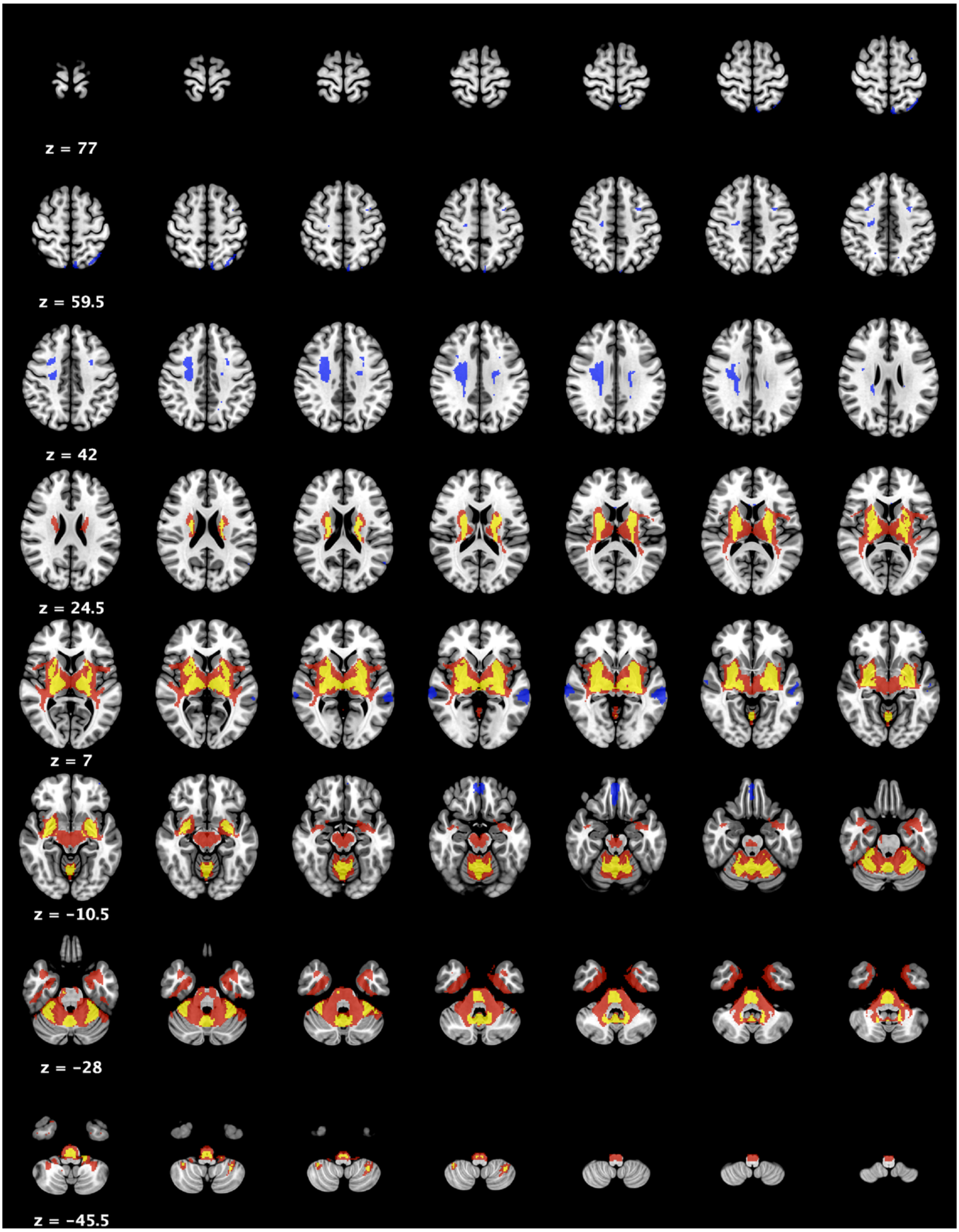

Supplementary File 5. General dystonia network. Axial slices showing brain regions surviving ≥3 of the four statistical tests used to define the general dystonia network. Red = 3/4 tests; yellow = 4/4 tests (positive connectivity). Blue = 3/4 tests (negative connectivity). No negatively connected voxels survived all 4 tests.

Supplementary File 6. Clusters of the general dystonia network. Max X, Y, Z refer to the location where the highest N of lesions were connected within the cluster, while COG X, Y, Z refers to its centre of gravity. Positively connected clusters surviving 4/4 statistical tests are shown. While no negatively connected clusters survived 4/4 tests, therefore clusters surviving 3/4 tests are shown.

Positive connectivity

	N voxels	MAX X (MNI)	MAX Y	MAX Z	COG X (MNI)	COG Y	COG Z
R basal ganglia, thalamus, claustrum	2308	18	-10	4	23	-8	3
L basal ganglia, thalamus, claustrum	2350	-16	-22	8	-23	-10	3
Cerebellum	2458	-34	-44	-30	-2	-55	-26
Brainstem	411	4	-30	-46	2	-28	-41

Negative connectivity

	N voxels	MAX X (MNI)	MAX Y	MAX Z	COG X (MNI)	COG Y	COG Z
R subcortical WM	792	22	0	32	25	-10	36
L mid/superior termporal gyrus	286	-66	-30	-2	-61	-31	-2
Medial frontal gyrus	146	-2	50	-20	1	48	-20
R mid/superior termporal gyrus	106	66	-28	-2	65	-26	-1
L subcortical WM	98	-24	-8	34	-22	-14	34
L subcortical WM/middle frontal gyrus	72	-38	6	54	-30	6	44
L medial parietal lobe	71	-10	-76	60	-7	-74	58
L superior parietal lobe	52	-34	-64	60	-33	-65	60

Network connectivity and extent of dystonia in the body – number of overlapping voxels

We investigated whether lesion connectivity to this dystonia brain network could predict a greater extent dystonia in the body. Results for connectivity strength and its association to expression of dystonia in the body are shown in the main manuscript. We also investigated whether the number of overlapping voxels predicted the extent of dystonia in the body. To calculate this, individual lesion network maps were binarised at a t-value of ≥7 (i.e., the same binarisation process and t-value as in the overlap analysis, described in manuscript) and the number of binarised voxels that hit/overlapped with the general dystonia network were extracted for analysis. These values were then associated with: 1) the number of body parts affected by dystonia (Spearman rank correlation); and 2) dystonia body distribution (multiple linear regression with post-hoc pairwise tests where regression showed significance). Analyses demonstrated that the number of voxels from a case's lesion network map that hit the general dystonia network was significantly associated with the number of body parts affected by dystonia ($\rho=0.229$; $p=0.002$) and dystonia body distribution ($F[4,174]=3.13$; $p=0.016$) (Figure).

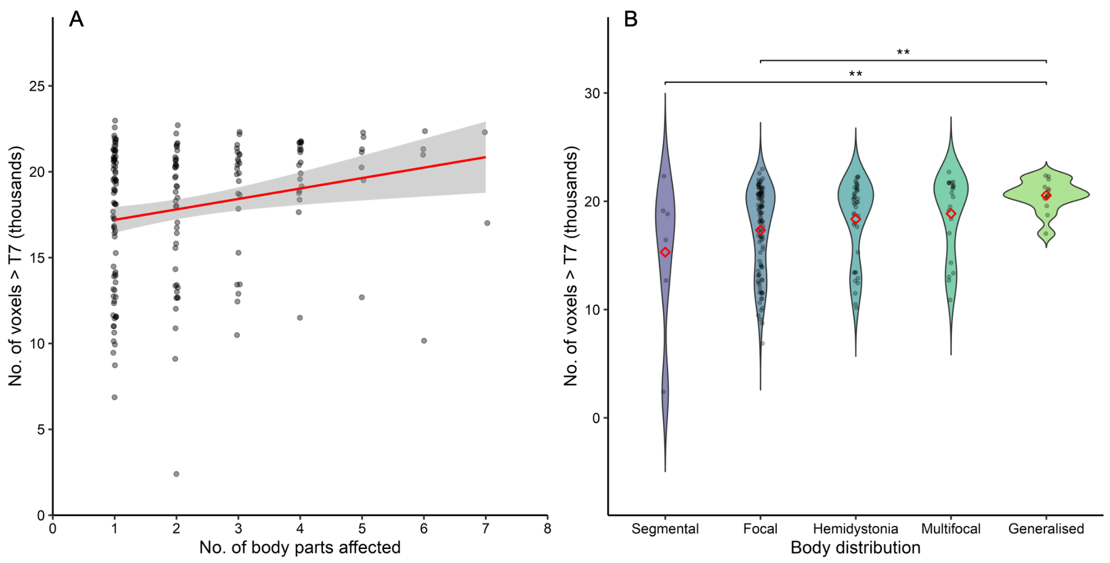

Supplementary File 7. Network connectivity and extent of dystonia in the body. There was a significant positive association between the number of voxels hitting the general dystonia network and the number of body parts affected by dystonia (Figure A, p=0.002). Similarly, patients with generalised dystonia had a significantly higher number of voxels hitting the general dystonia network compared to patients with focal and segmental dystonia (Figure B). ** = p<0.01. T7 = t-value of 7.

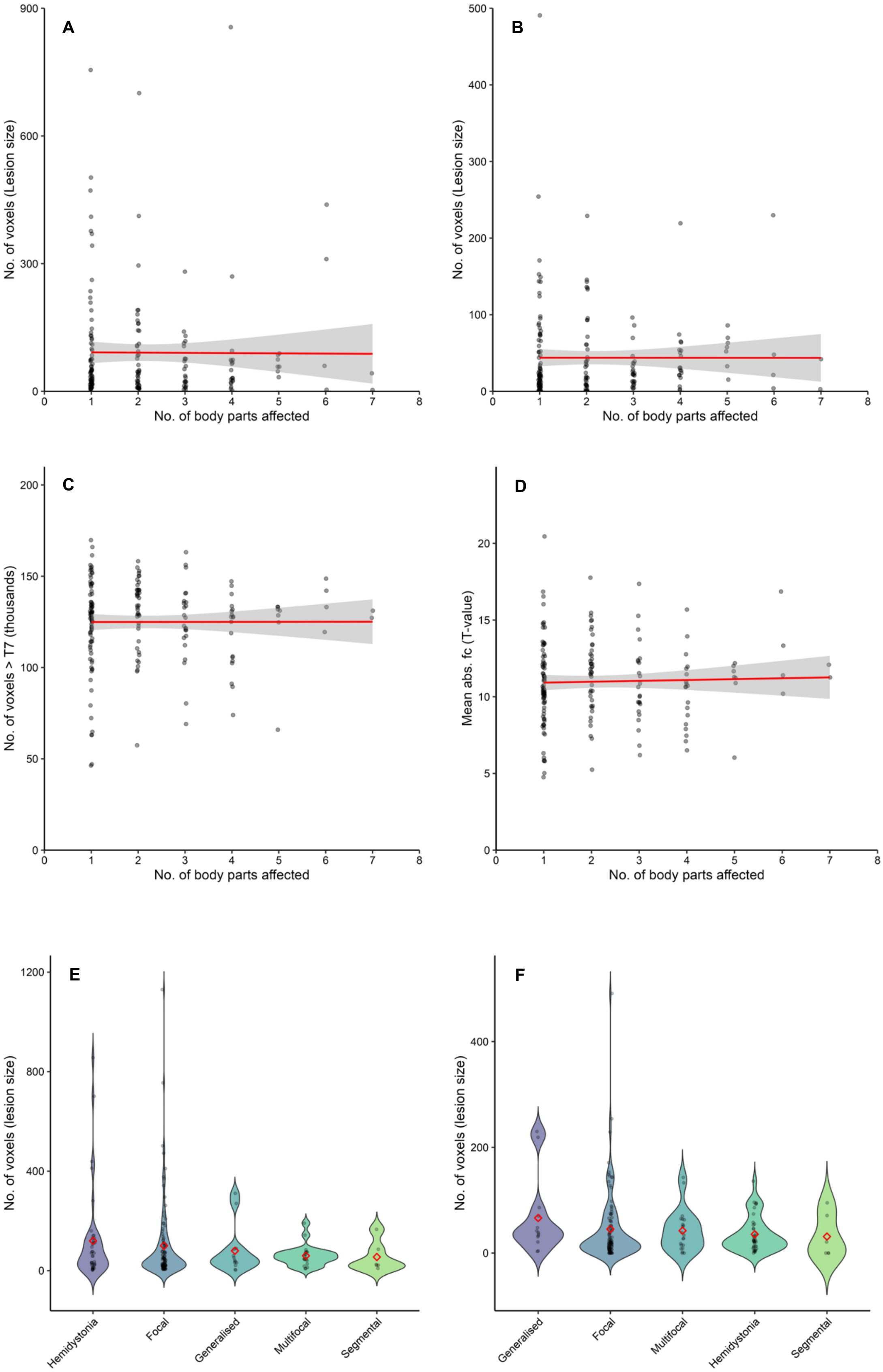
A
B
C
D
E
F
No. of voxels (Lesion size)
No. of body parts affected
No. of voxels > T7 (thousands)
Mean abs. fc (T-value)
No. of voxels (lesion size)
Hemidystonia
Focal
Generalised
Multifocal
Segmental

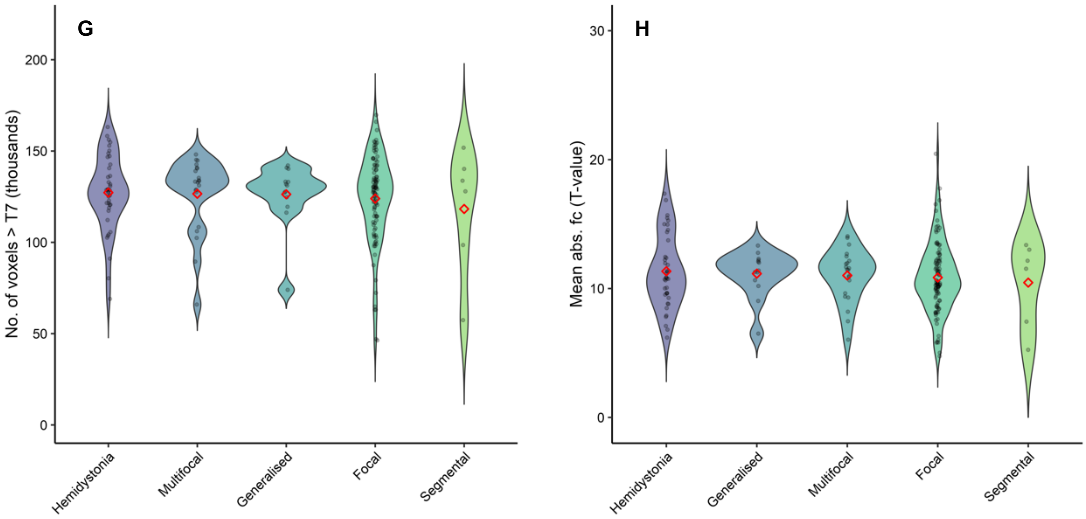

Supplementary file 8. Network connectivity and extent of dystonia in the body. Figure 3 in the main manuscript demonstrated that for each case's lesion network map, there were positive associations between the involvement of the general dystonia network and the extent of dystonia in the body. These analyses were not significant for lesion size or for whole brain analyses, shown here (all relationships p>0.20). A) (Spearman) correlation between lesion size and number of body parts affected, whole brain. B) Correlation between lesion size and number of body parts affected, general dystonia network. C) Correlation between number of voxels ≥T7 from a case's lesion network map and number of body parts affected, whole brain. D) Correlation between mean absolute functional connectivity of T-maps and number of body parts affected, whole brain. E) Regression analysis of lesion size between the dystonia body distributions, whole brain. F) Regression analysis of lesion size between the dystonia body distributions, general dystonia network. G) Regression analysis of number of voxels ≥T7 from a case's lesion network map between the dystonia body distributions, whole brain. H) Regression analysis of mean absolute functional between dystonia body distributions, whole brain.

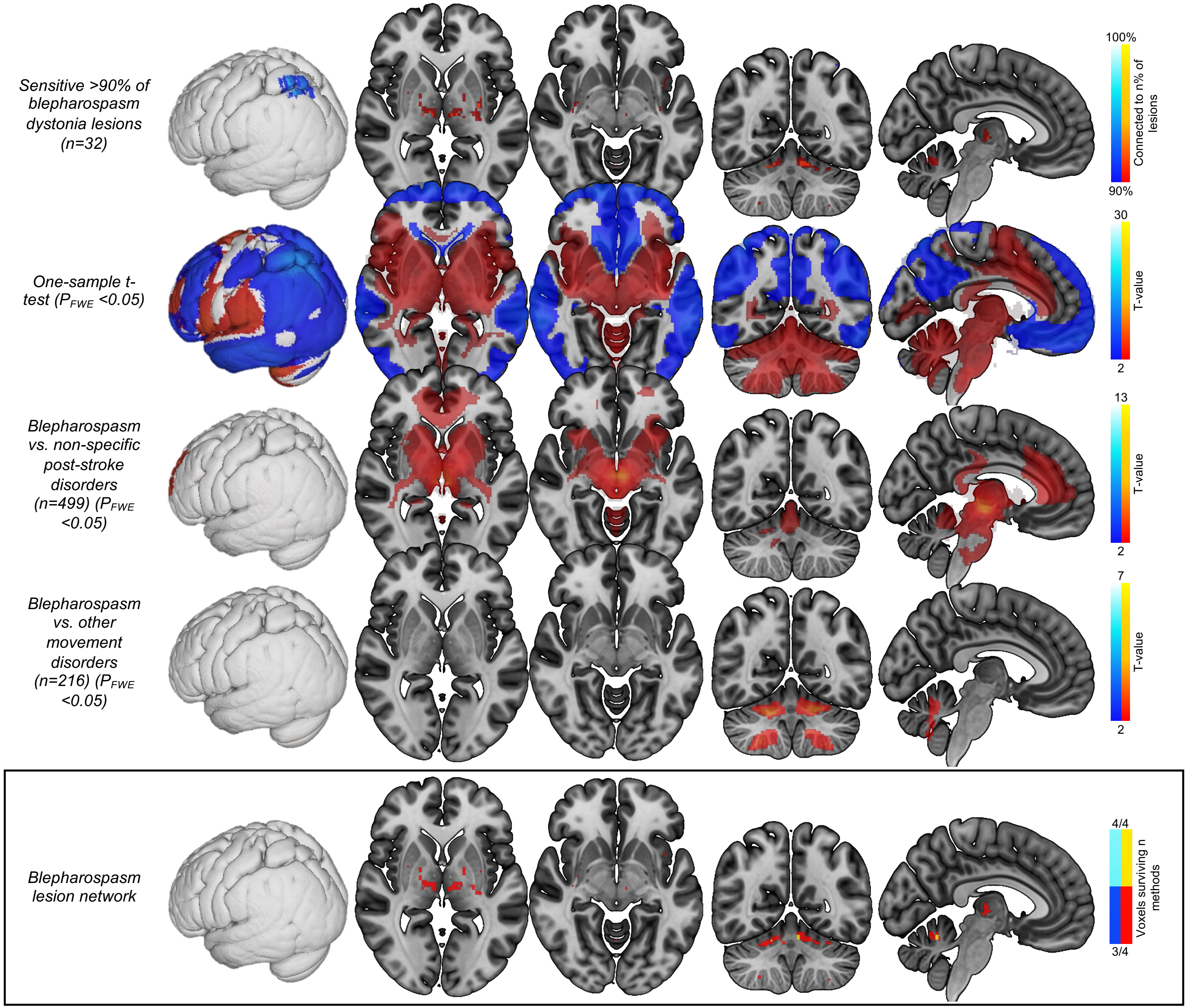

Supplementary file 9. Body part and distribution lesion networks. Figure shows the individual tests that were used to define each of the body part and body distribution lesion networks.

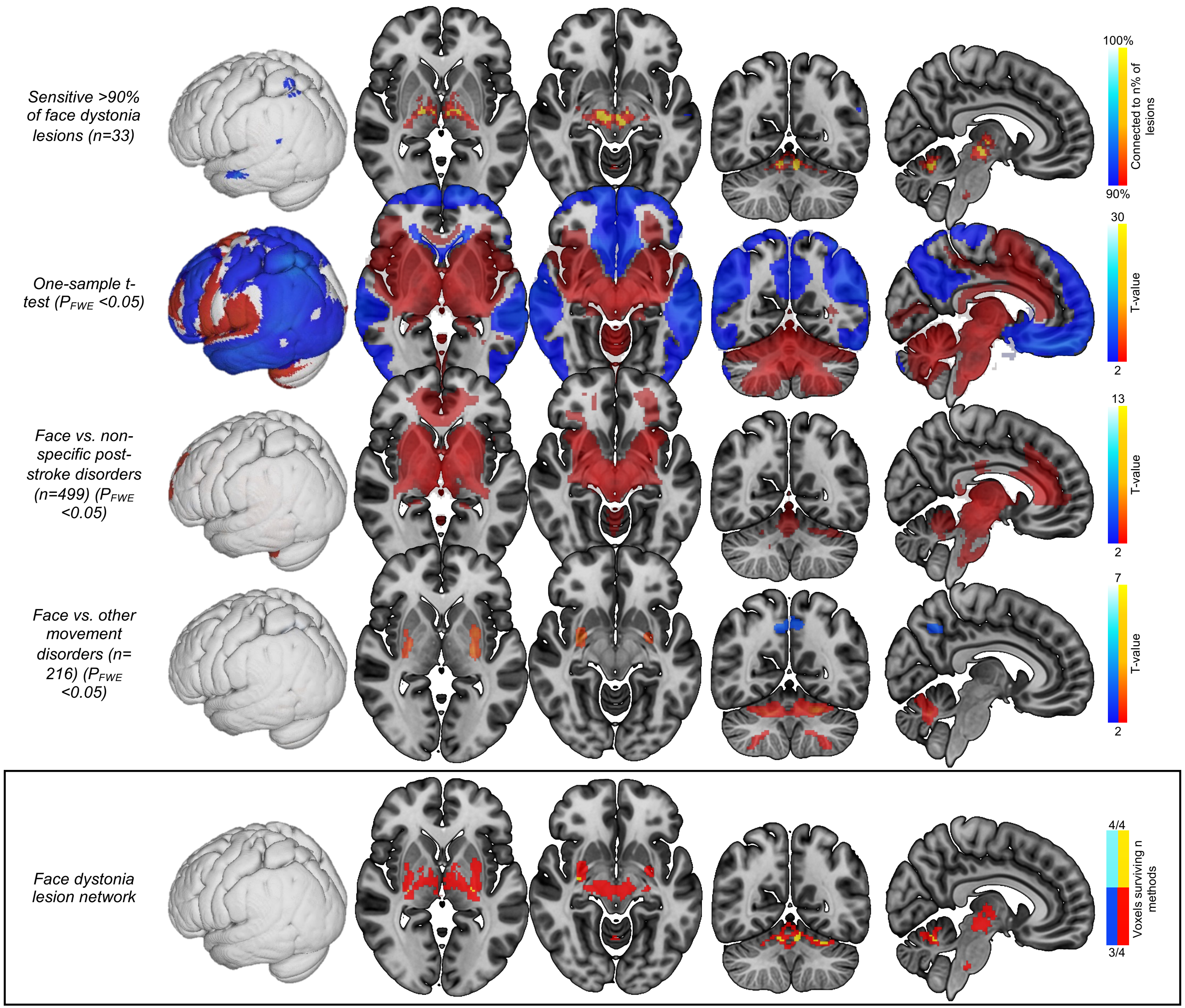
Sensitive >90% of face dystonia lesions (n=33)
One-sample t-test (P_{FWE} <0.05)
Face vs. non-specific post-stroke disorders (n=499) (P_{FWE} <0.05)
Face vs. other movement disorders (n= 216) (P_{FWE} <0.05)
Face dystonia lesion network
100%
90%
Connected to n% of lesions
30
2
T-value
13
2
T-value
7
2
T-value
4/4
3/4
Voxels surviving n methods

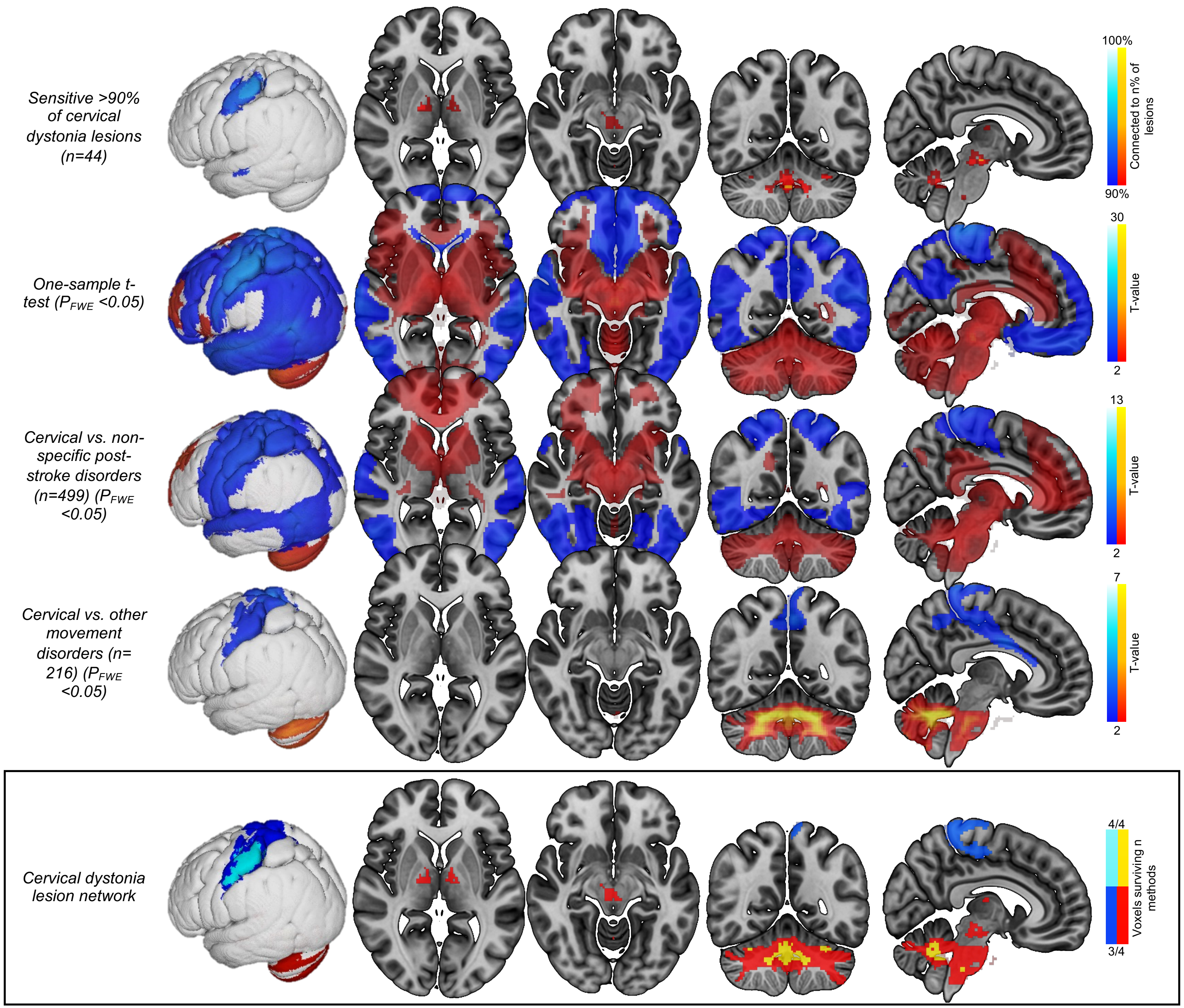
Sensitive >90% of cervical dystonia lesions (n=44)
100%
Connected to n% of lesions
90%
One-sample t-test (P_FWE <0.05)
30
T-value
2
Cervical vs. non-specific post-stroke disorders (n=499) (P_FWE <0.05)
13
T-value
2
Cervical vs. other movement disorders (n=216) (P_FWE <0.05)
7
T-value
2
Cervical dystonia lesion network
4/4
Voxels surviving n methods
3/4

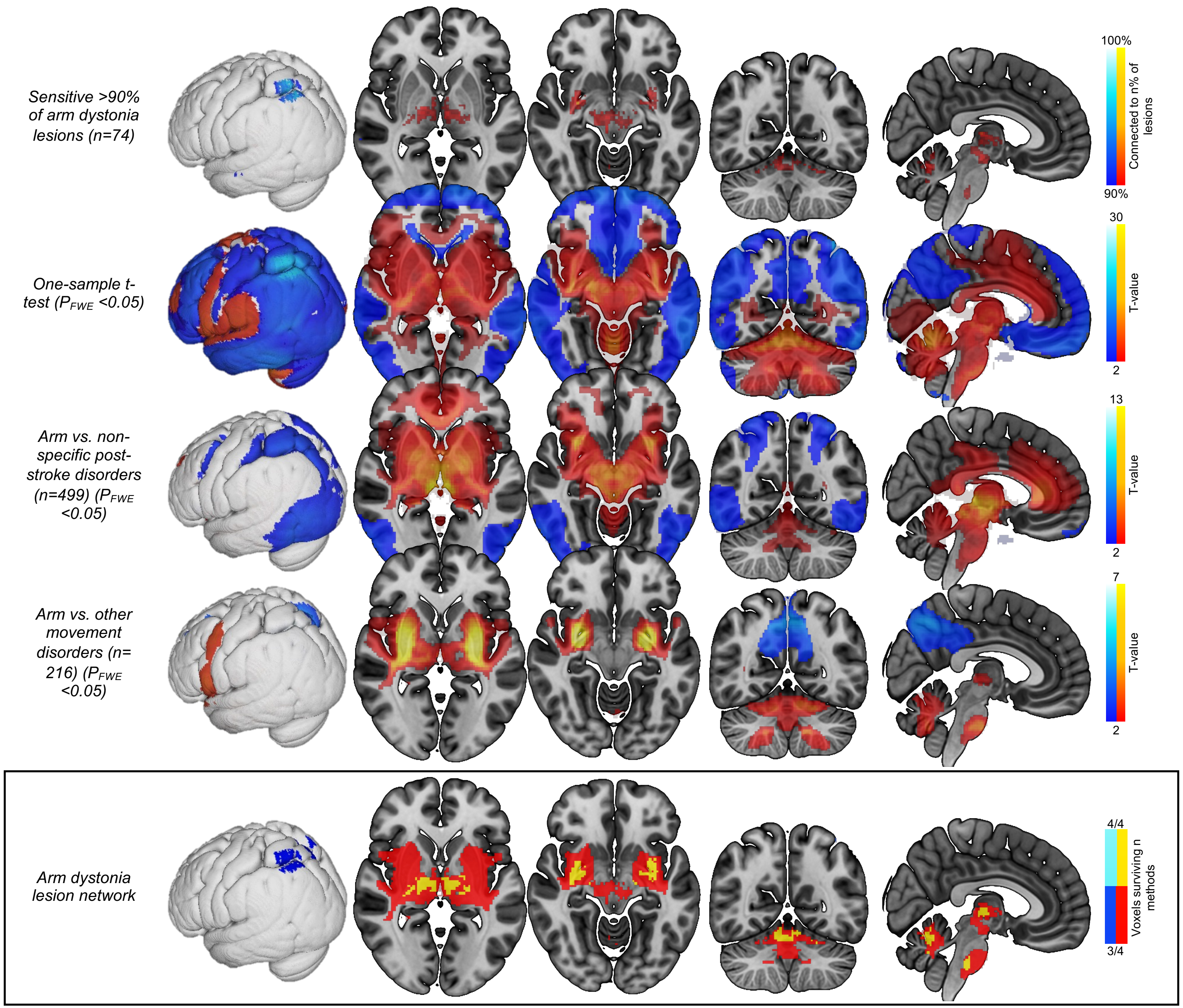
Sensitive >90% of arm dystonia lesions (n=74)
One-sample t-test (PFWE <0.05)
Arm vs. non-specific post-stroke disorders (n=499) (PFWE <0.05)
Arm vs. other movement disorders (n= 216) (PFWE <0.05)
Arm dystonia lesion network
100%
Connected to n% of lesions
90%
30
T-value
2
13
T-value
2
7
T-value
2
4/4
Voxels surviving n methods
3/4

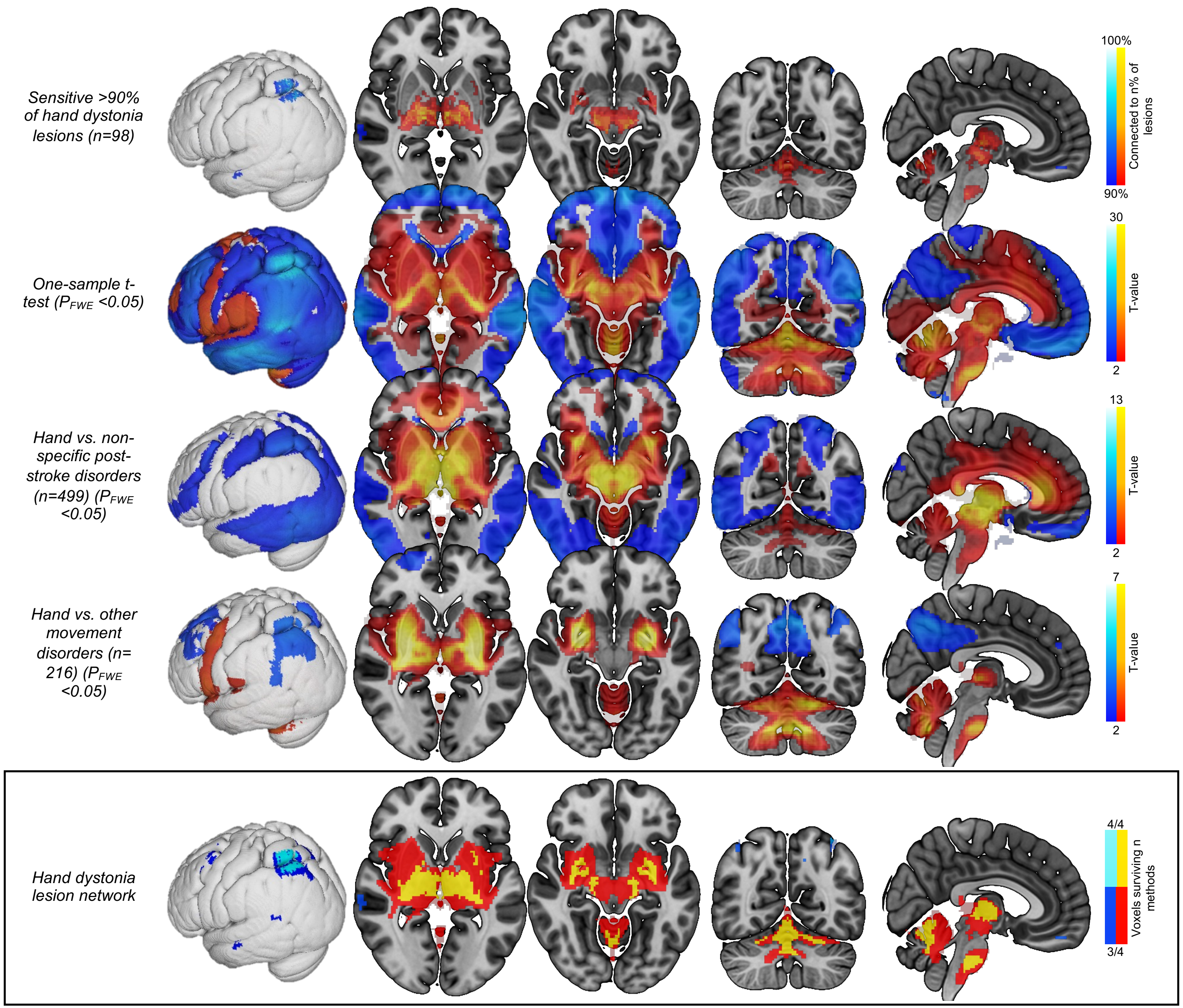
Sensitive >90% of hand dystonia lesions (n=98)
One-sample t-test (PFWE <0.05)
Hand vs. non-specific post-stroke disorders (n=499) (PFWE <0.05)
Hand vs. other movement disorders (n=216) (PFWE <0.05)
Hand dystonia lesion network
100%
90%
Connected to n% of lesions
30
2
T-value
13
2
T-value
7
2
T-value
4/4
3/4
Voxels surviving n methods

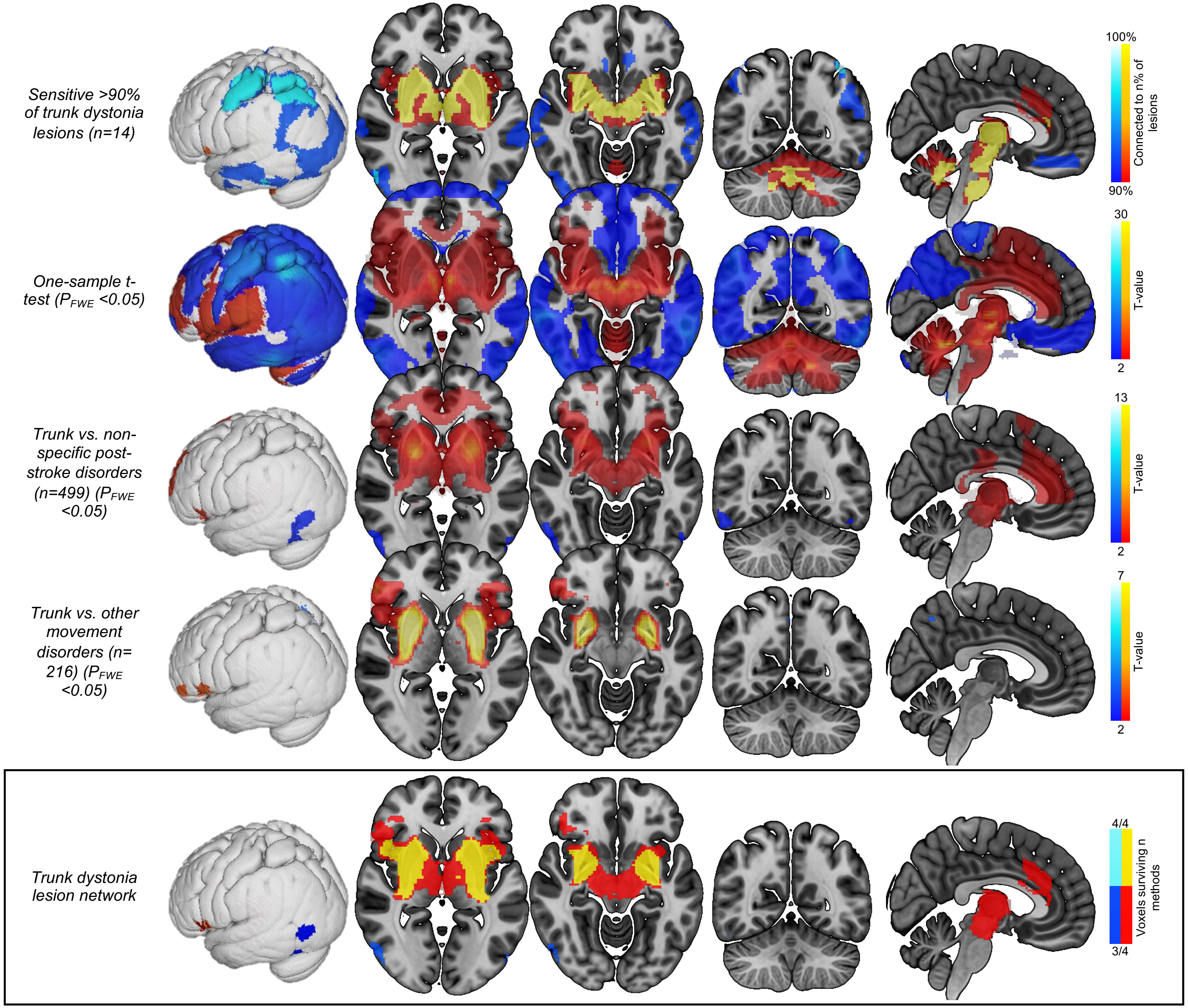
Sensitive >90% of trunk dystonia lesions (n=14)
One-sample t-test (P_{FWE} <0.05)
Trunk vs. non-specific post-stroke disorders (n=499) (P_{FWE} <0.05)
Trunk vs. other movement disorders (n= 216) (P_{FWE} <0.05)
Trunk dystonia lesion network
100%
Connected to n% of lesions
90%
30
T-value
2
13
T-value
2
7
T-value
2
4/4
Voxels surviving n methods
3/4

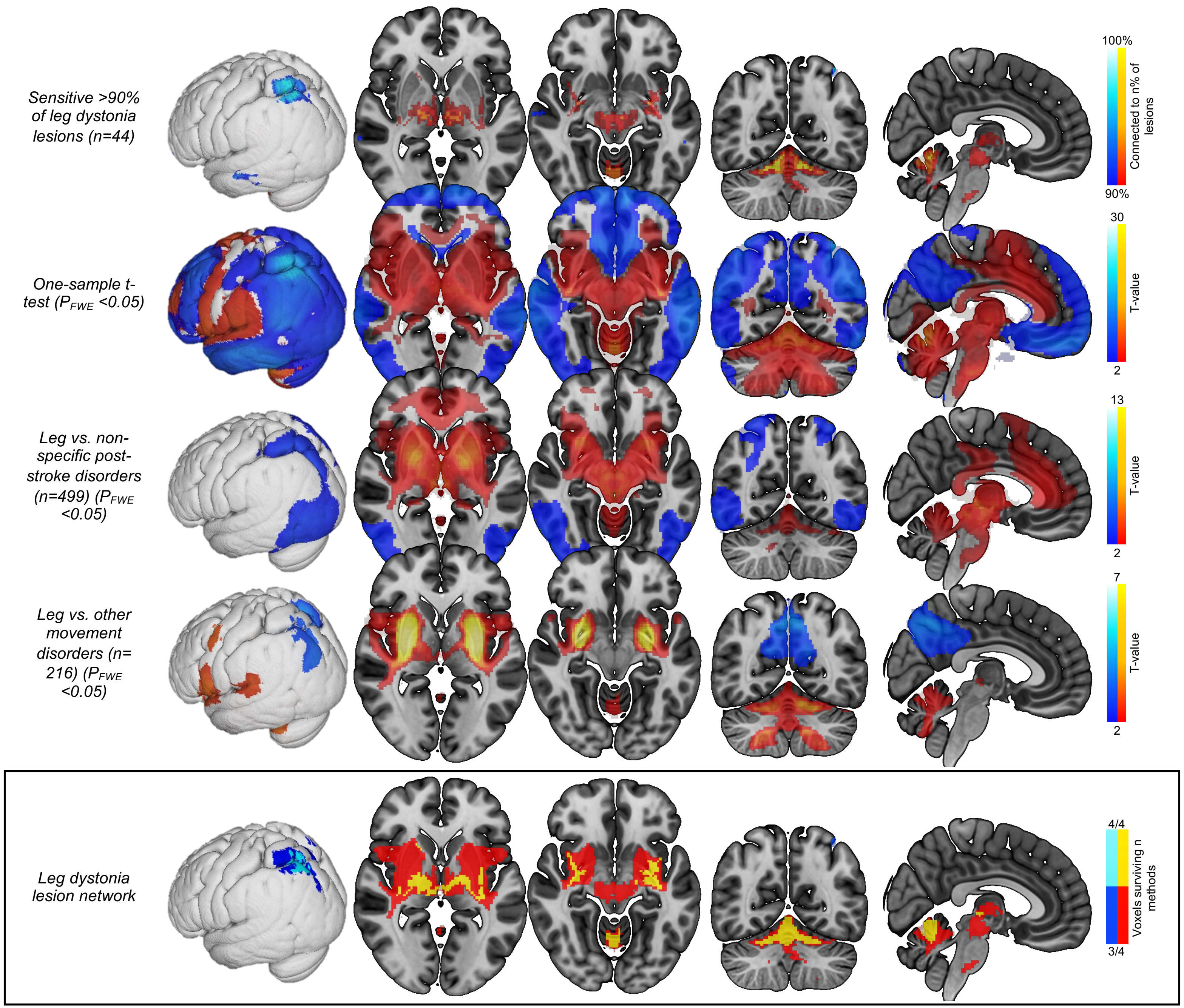

Sensitive >90% of leg dystonia lesions (n=44)
One-sample t-test (P_{FWE} <0.05)
Leg vs. non-specific post-stroke disorders (n=499) (P_{FWE} <0.05)
Leg vs. other movement disorders (n= 216) (P_{FWE} <0.05)
Leg dystonia lesion network
100%
Connected to n% of lesions
90%
30
T-value
2
13
T-value
2
7
T-value
2
4/4
Voxels surviving n methods
3/4

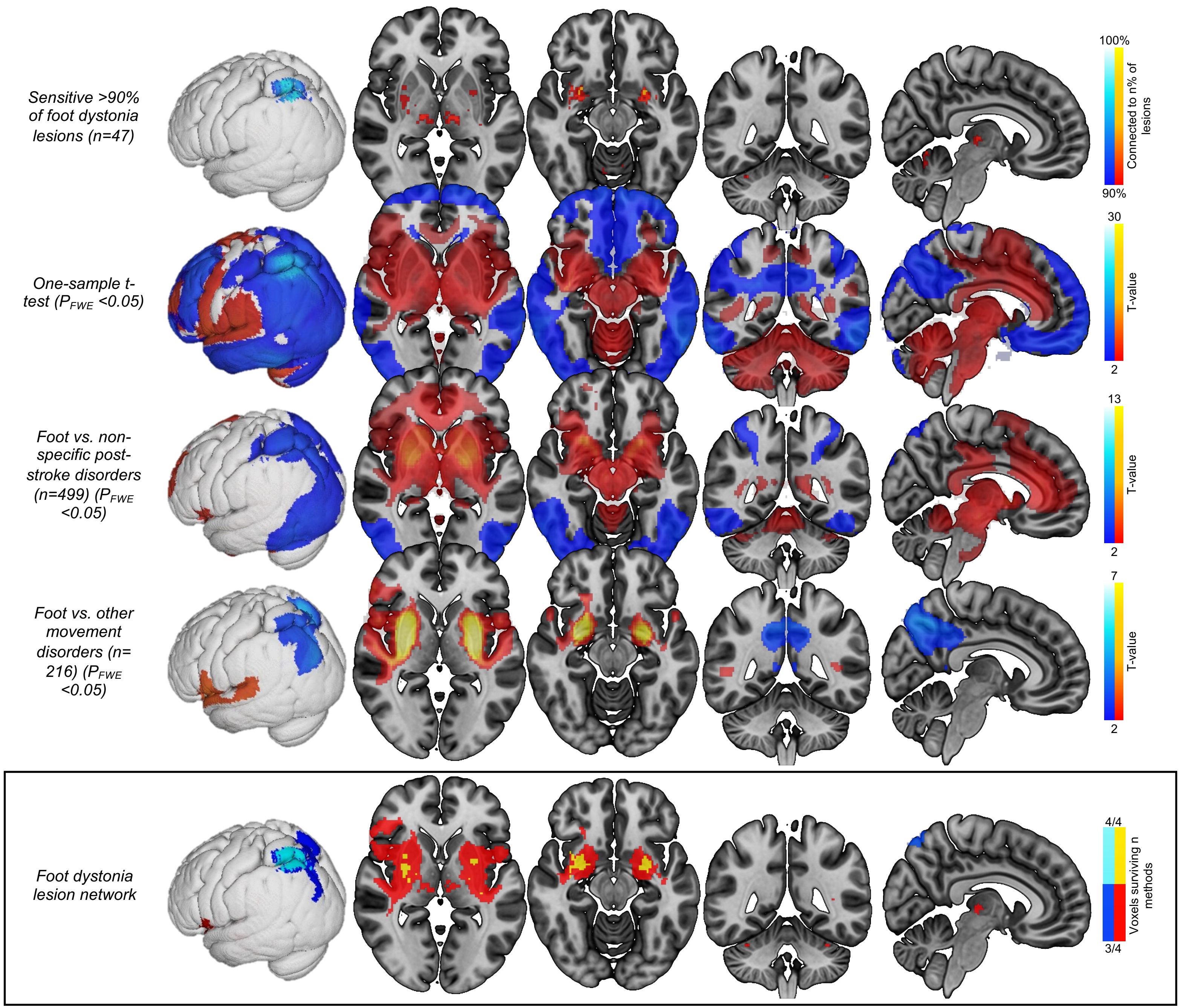

Sensitive >90% of foot dystonia lesions (n=47)
One-sample t-test (PFWE <0.05)
Foot vs. non-specific post-stroke disorders (n=499) (PFWE <0.05)
Foot vs. other movement disorders (n= 216) (PFWE <0.05)
Foot dystonia lesion network
100%
Connected to n% of lesions
90%
30
T-value
2
13
T-value
2
7
T-value
2
4/4
Voxels surviving n methods
3/4

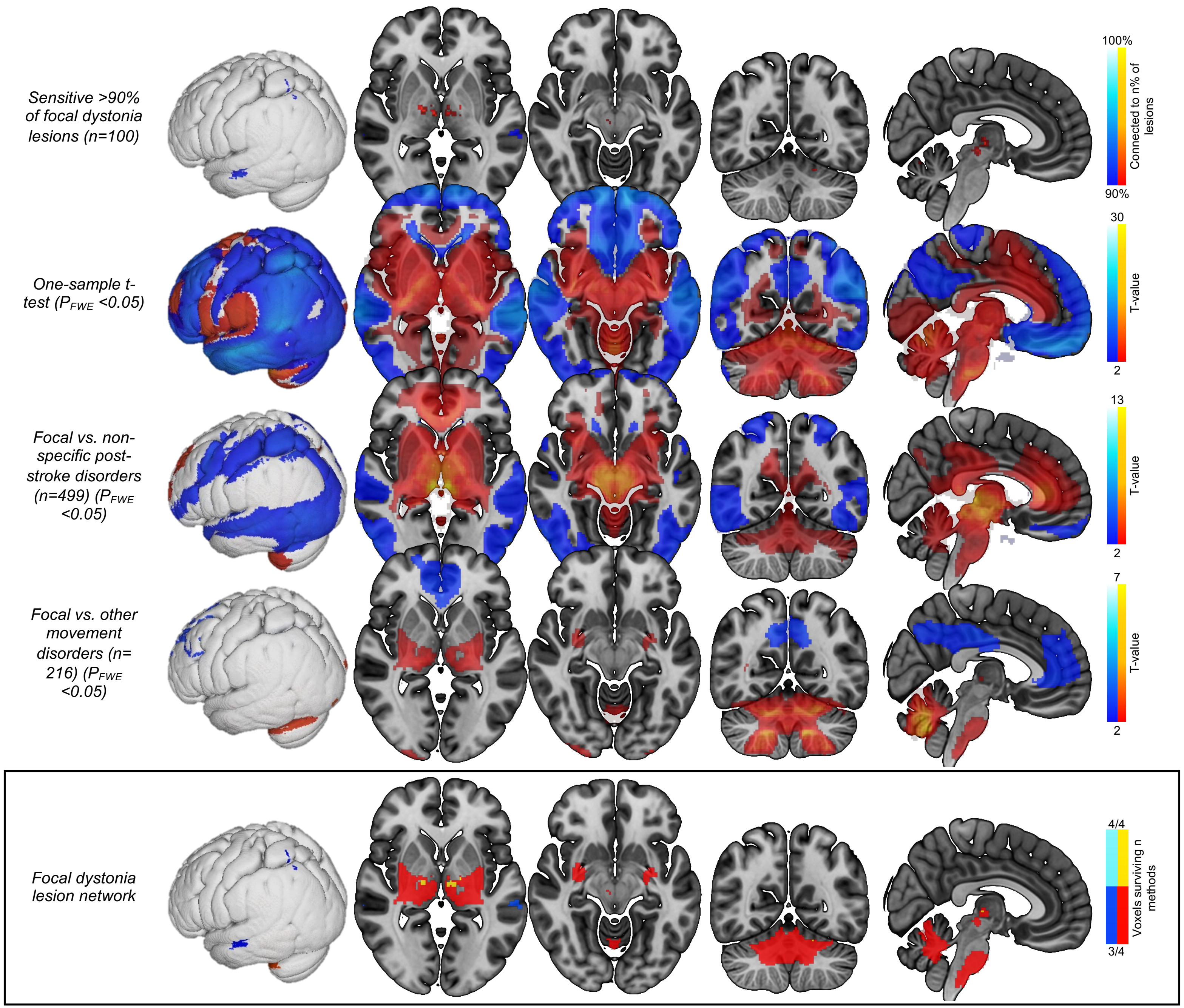

Sensitive >90% of focal dystonia lesions (n=100)
One-sample t-test (P_{FWE} <0.05)
Focal vs. non-specific post-stroke disorders (n=499) (P_{FWE} <0.05)
Focal vs. other movement disorders (n=216) (P_{FWE} <0.05)
Focal dystonia lesion network
100%
Connected to n% of lesions
90%
30
T-value
2
13
T-value
2
7
T-value
2
4/4
Voxels surviving n methods
3/4

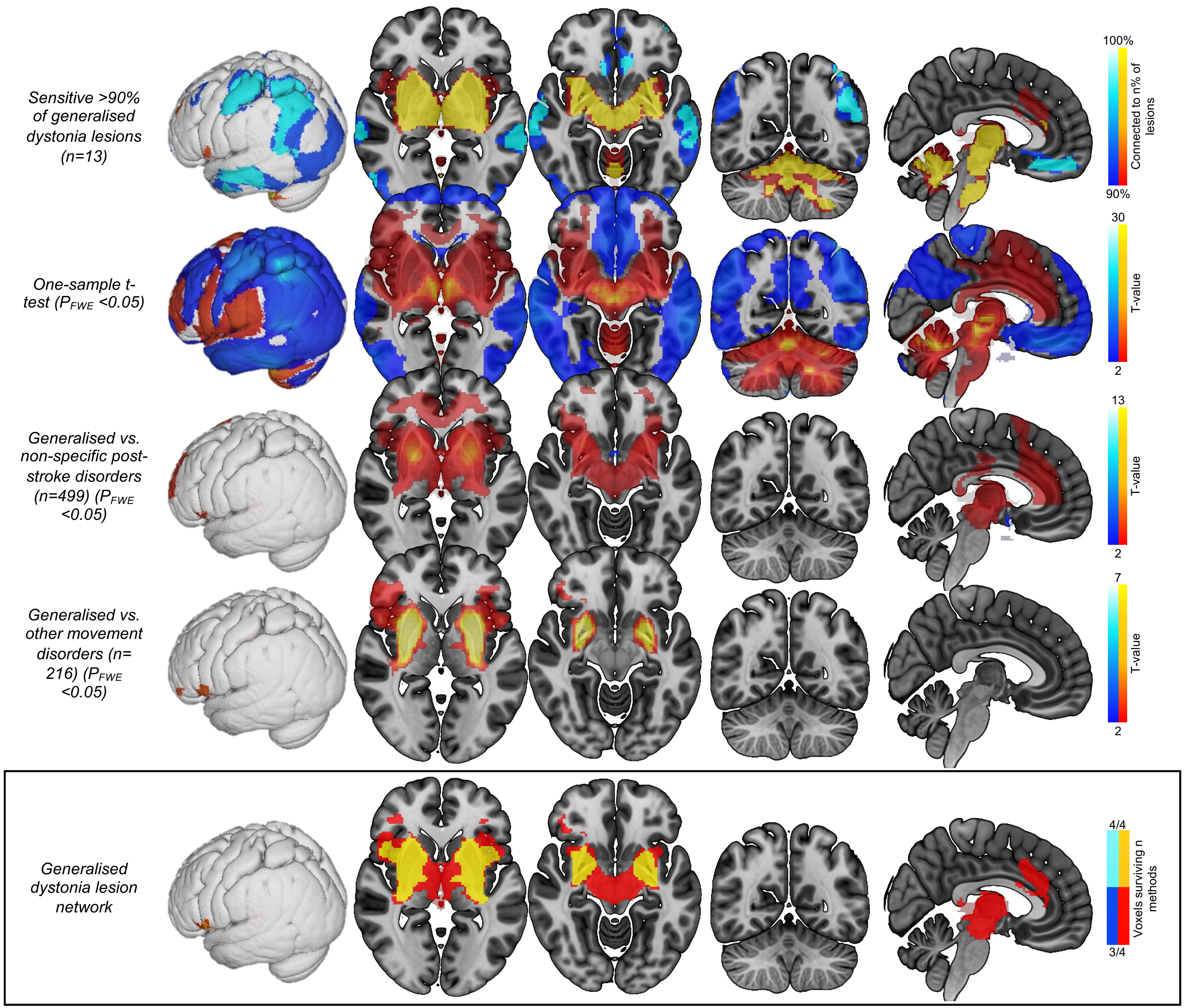

Sensitive >90% of generalised dystonia lesions (n=13)
One-sample t-test (P_{FWE} <0.05)
Generalised vs. non-specific post-stroke disorders (n=499) (P_{FWE} <0.05)
Generalised vs. other movement disorders (n=216) (P_{FWE} <0.05)
Generalised dystonia lesion network
100%
Connected to n% of lesions
90%
30
T-value
2
13
T-value
2
7
T-value
2
4/4
Voxels surviving n methods
3/4

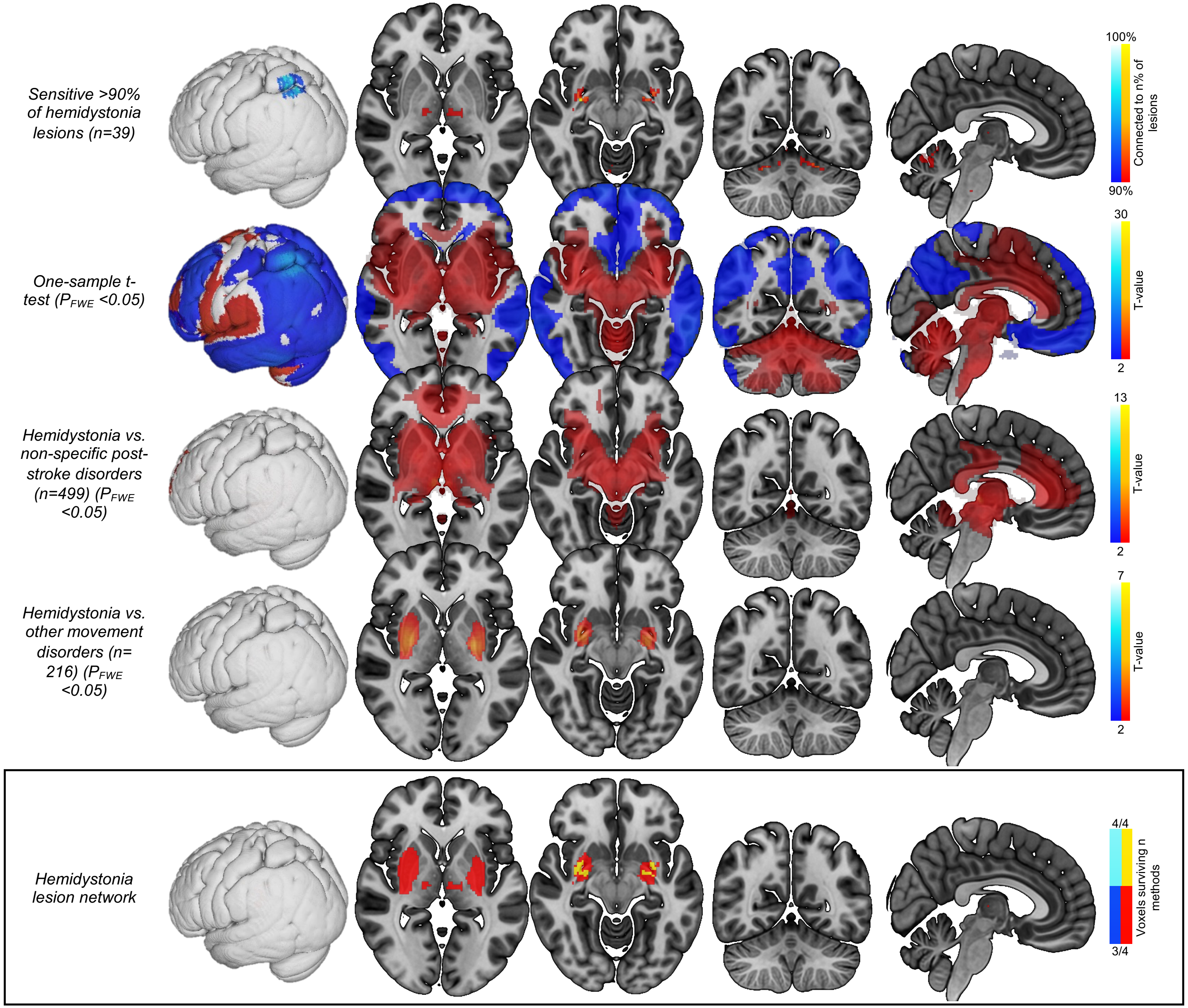

Sensitive >90% of hemidystonia lesions (n=39)
One-sample t-test (PFWE <0.05)
Hemidystonia vs. non-specific post-stroke disorders (n=499) (PFWE <0.05)
Hemidystonia vs. other movement disorders (n=216) (PFWE <0.05)
Hemidystonia lesion network
100%
Connected to n% of lesions
90%
30
T-value
2
13
T-value
2
7
T-value
2
4/4
Voxels surviving n methods
3/4

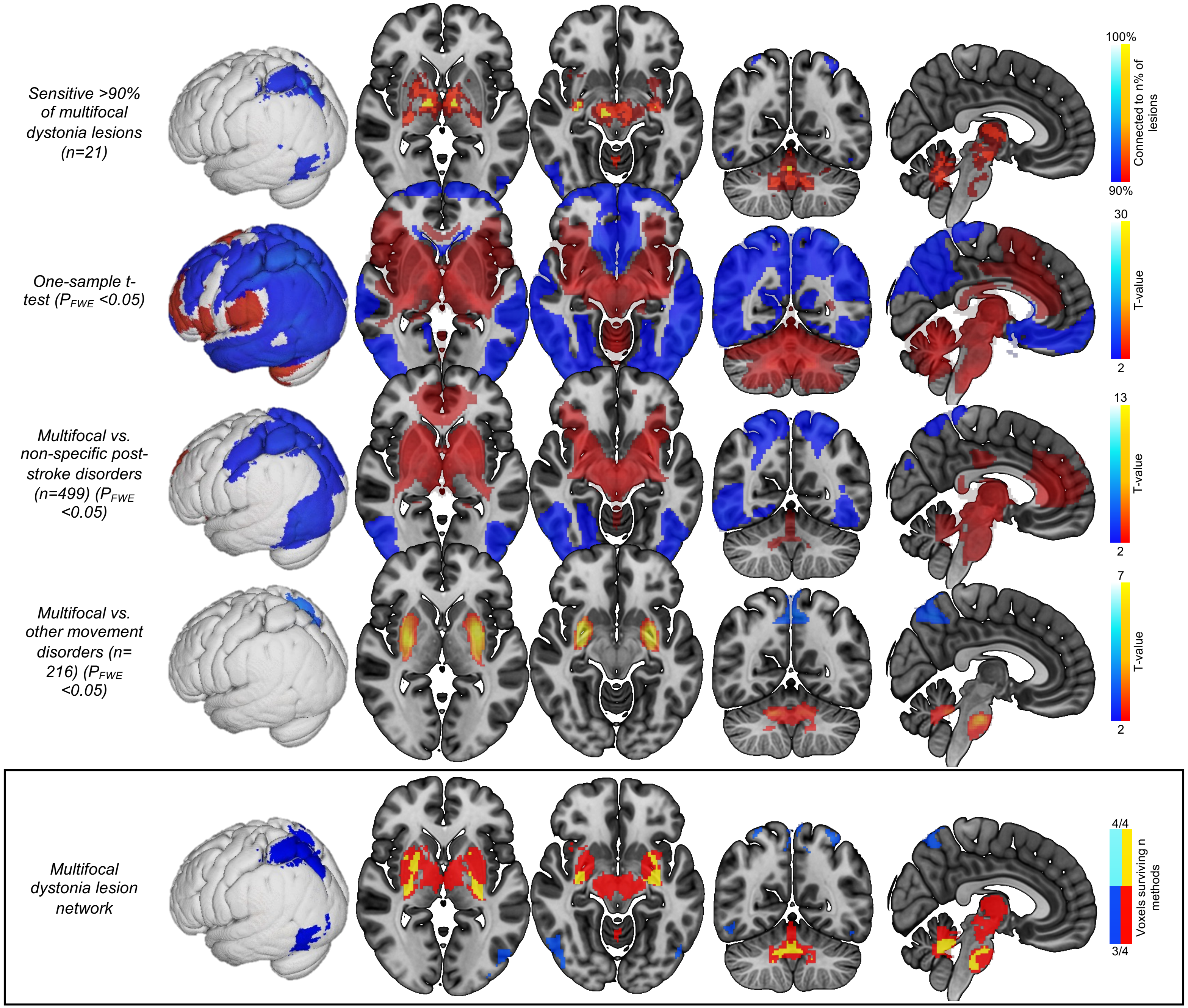
Sensitive >90% of multifocal dystonia lesions (n=21)
One-sample t-test (P_{FWE} <0.05)
Multifocal vs. non-specific post-stroke disorders (n=499) (P_{FWE} <0.05)
Multifocal vs. other movement disorders (n= 216) (P_{FWE} <0.05)
Multifocal dystonia lesion network
100%
Connected to n% of lesions
90%
30
T-value
2
13
T-value
2
7
T-value
2
4/4
Voxels surviving n methods
3/4

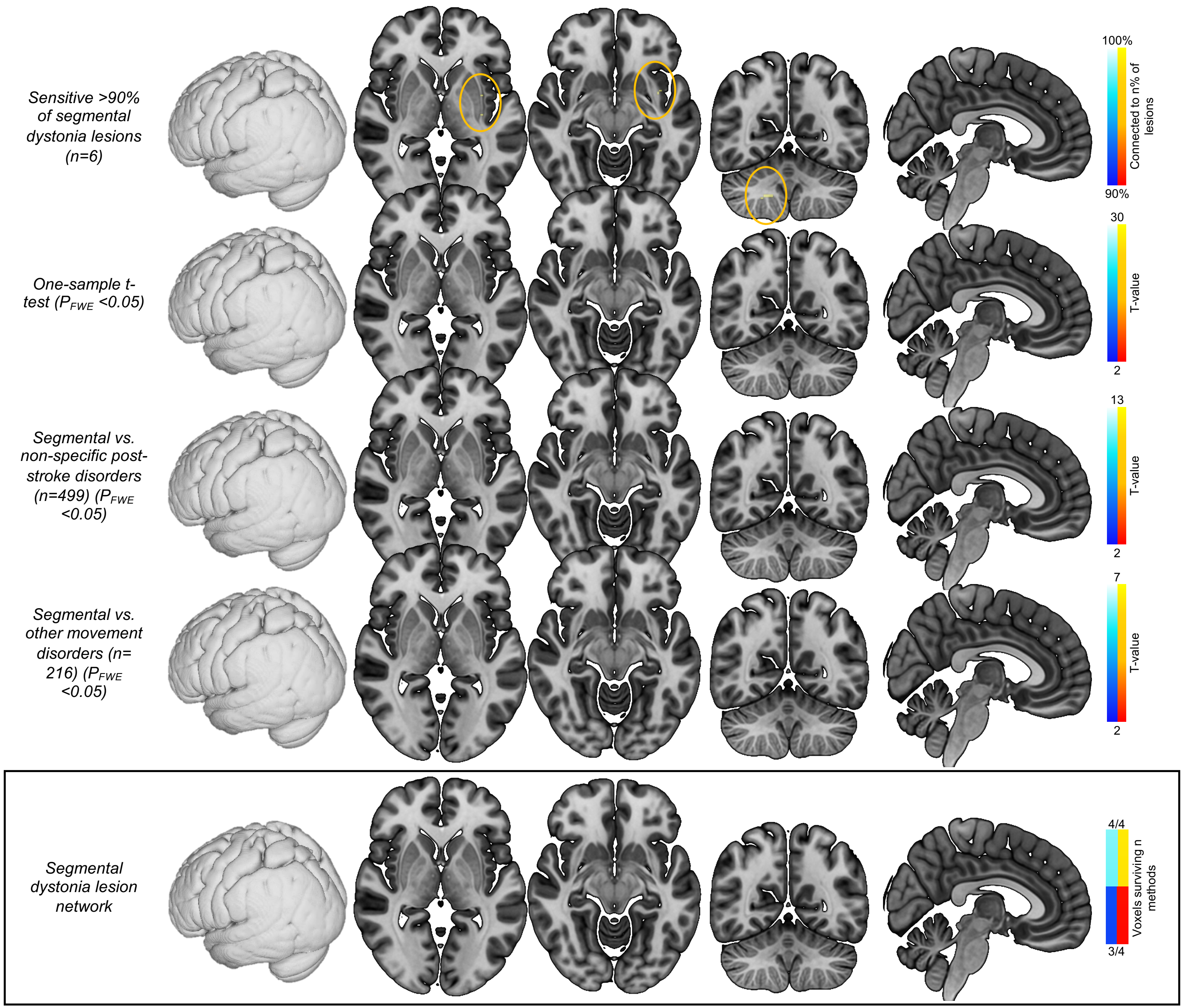
Sensitive >90% of segmental dystonia lesions (n=6)
One-sample t-test (P_{FWE} <0.05)
Segmental vs. non-specific post-stroke disorders (n=499) (P_{FWE} <0.05)
Segmental vs. other movement disorders (n= 216) (P_{FWE} <0.05)
Segmental dystonia lesion network
100%
90%
Connected to n% of lesions
30
2
T-value
13
2
T-value
7
2
T-value
4/4
3/4
Voxels surviving n methods

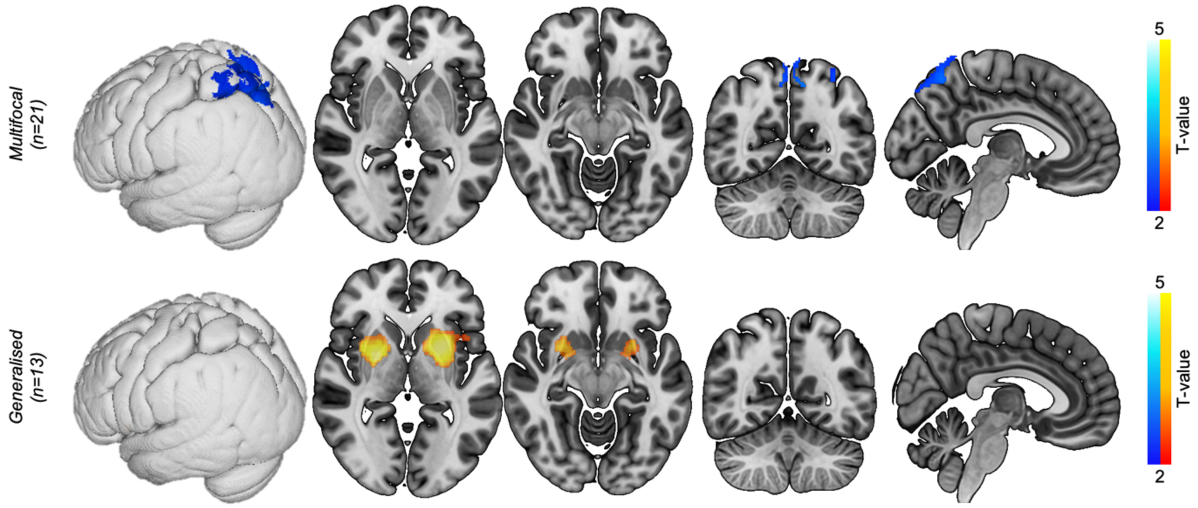

Supplementary File 10. Significant differences between dystonias - body distribution. We investigated whether there were significant differences in lesion connectivity between body distributions. Here, each dystonia body distribution was compared to other dystonia cases without that that body distribution. The generalised and trunk dystonia findings were similar given that 13/14 cases with trunk dystonia were categorised as having generalised dystonia. T-values are shown for visual purposes, masked to regions p<0.05 FWE corrected. Body distributions not shown did not demonstrate significant differences.

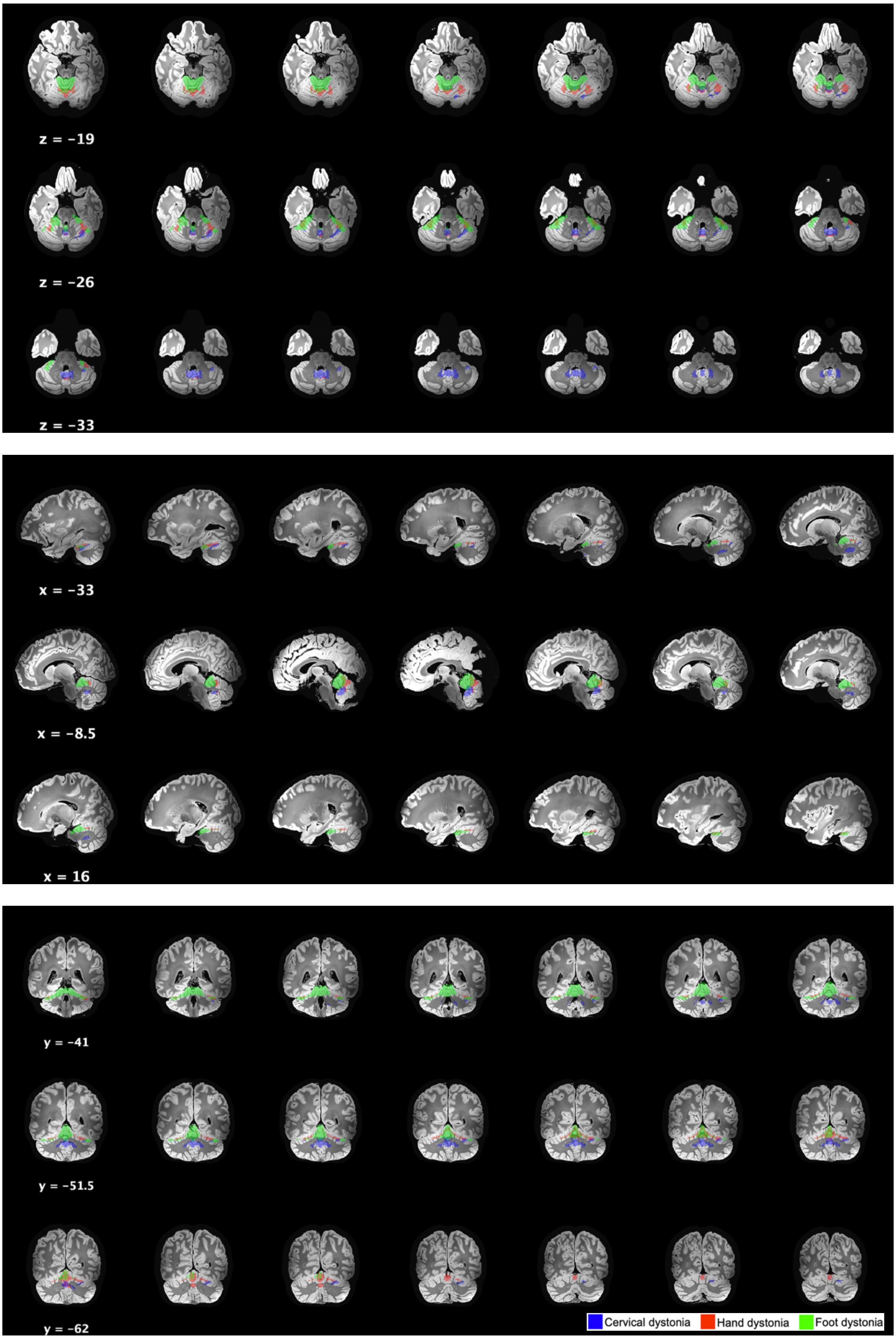

Supplementary File 11. Additional slices of cervical, hand, and foot dystonia lesion networks in the cerebellum.

Supplementary File 12. Number of voxels of the cervical, hand, and foot dystonia network falling within the sub-structures of the SUIT atlas of the cerebellum. Sub-structures not shown did not contain at least 10 network voxels or have voxels cover at least 10% of the atlas region.

**<u>Cervical dystonia</u>**	***Network voxels***	***Total voxels of structure***	***% of atlas region implicated***
Left VI	147	1561	9.4
Vermis IX	81	128	63.3
Left Dentate	76	250	30.4
Right White	38	1018	3.7
Left IX	34	604	5.6
Vermis X	33	59	55.9
Right Dentate	29	255	11.4
Left White	28	1069	2.6
Right IX	27	593	4.6
Vermis VIIIa	18	194	9.3
Vermis VI	17	358	4.7
Left Interposed	11	38	28.9
Right Interposed	10	38	26.3
Right Fastigial	6	7	85.7
Left Fastigial	5	6	83.3
**<u>Hand dystonia</u>**			
Left VI	219	1561	14.0
Right V	167	738	22.6
Left V	164	779	21.1
Right VI	110	1417	7.8
Vermis VI	83	358	23.2
Vermis VIIIa	22	194	11.3
Left I IV	17	632	2.7
Right I IV	13	663	2.0
**<u>Foot dystonia</u>**			
Right I IV	582	663	87.8
Left I IV	515	632	81.5
Right V	277	738	37.5
Left V	230	779	29.5
Right VI	151	1417	10.7
Left VI	106	1561	6.8
Left White	37	1069	3.5
Left CrusI	35	2210	1.6
Right CrusI	25	2230	1.1
Right White	24	1018	2.4
Right Fastigial	3	7	42.9
Left Fastigial	2	6	33.3

Supplementary File 13. Dystonia lesion network maps in the thalamus and putamen. Our primary analysis demonstrated that cervical, hand, and foot dystonia localised different sub-regions of the cerebellum (Figure 6, main manuscript). To test whether these findings were also present within other brain regions, we visualised these cervical, hand, and foot dystonia lesion network maps within the thalamus and the putamen, two other strongly localised structures in our analyses. Similar methods were used as in the cerebellar analysis. Specifically, cervical, hand, and foot lesion networks (Figure 4, main manuscript) were overlaid onto a standard adult brain, masked to the thalamus MNI structural probabilistic atlas, 25% probability) and putamen (Harvard-Oxford probabilistic subcortical atlas, 25% probability, and compared visually. As with the cerebellum analysis, the thresholds of these maps were adjusted to attempt to localise the major sub-region implicated, and to show a similar number of voxels between the groups, where possible. The following thresholds were used – **Thalamus** = Foot dystonia network map: voxels surviving ≥3 of 4 statistical tests, as shown in Figure 4; hand: 4/4; cervical: ≥3/4. **Putamen** = Foot: 4/4; hand: 4/4; cervical: ≥2/4.

Similar to the analysis within the cerebellum, there were differences in the sub-regions of the thalamus and putamen localised between cervical, hand, and foot dystonia (Figure). Specifically, compared to hand and foot dystonia, cervical dystonia preferentially mapped to superior and anterior portions of the thalamus, and superior and anterior portions of the putamen. Hand dystonia preferentially mapped to posterior regions of the putamen.

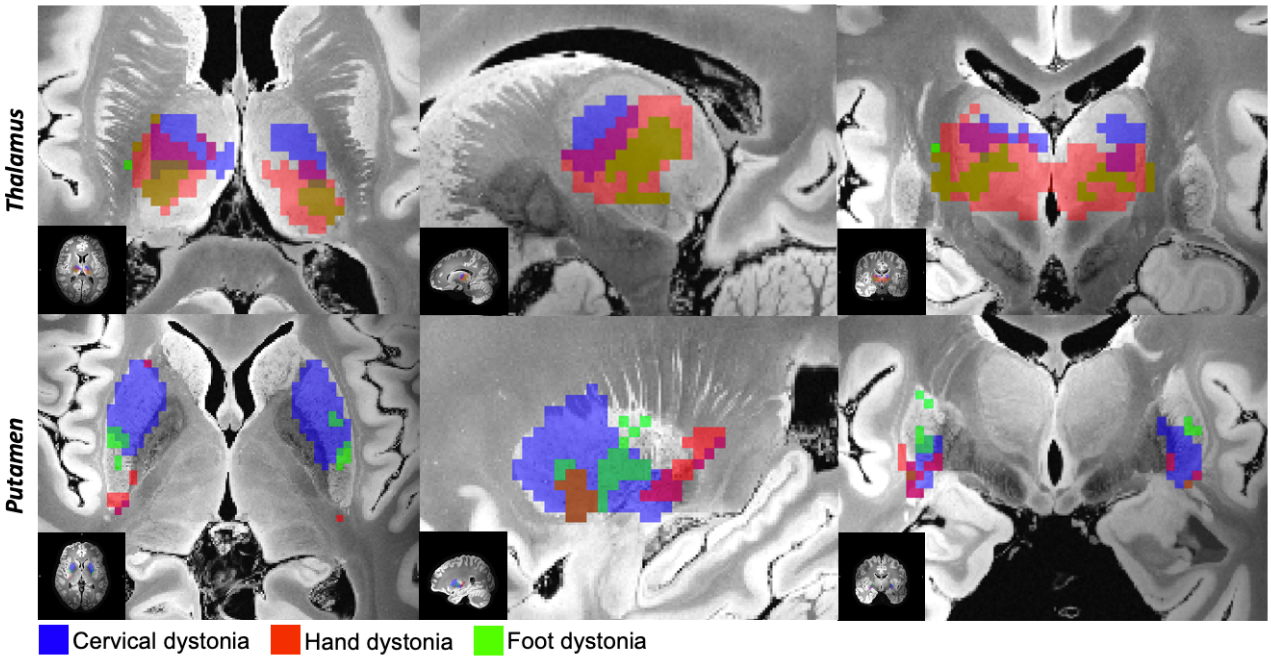

Supplementary File 13A. Dystonia lesion network maps in the thalamus and putamen. As with the cerebellum (Figure 7, main manuscript), cervical, hand, and foot dystonia lesion networks mapped to different sub-regions within the thalamus and putamen. Slices shown from L to R: Thalamus = Z: 11, X: -15, Y: -14.5. Putamen = Z: 1, X: 27.5, Y: -8.5. Additional slices are shown below.

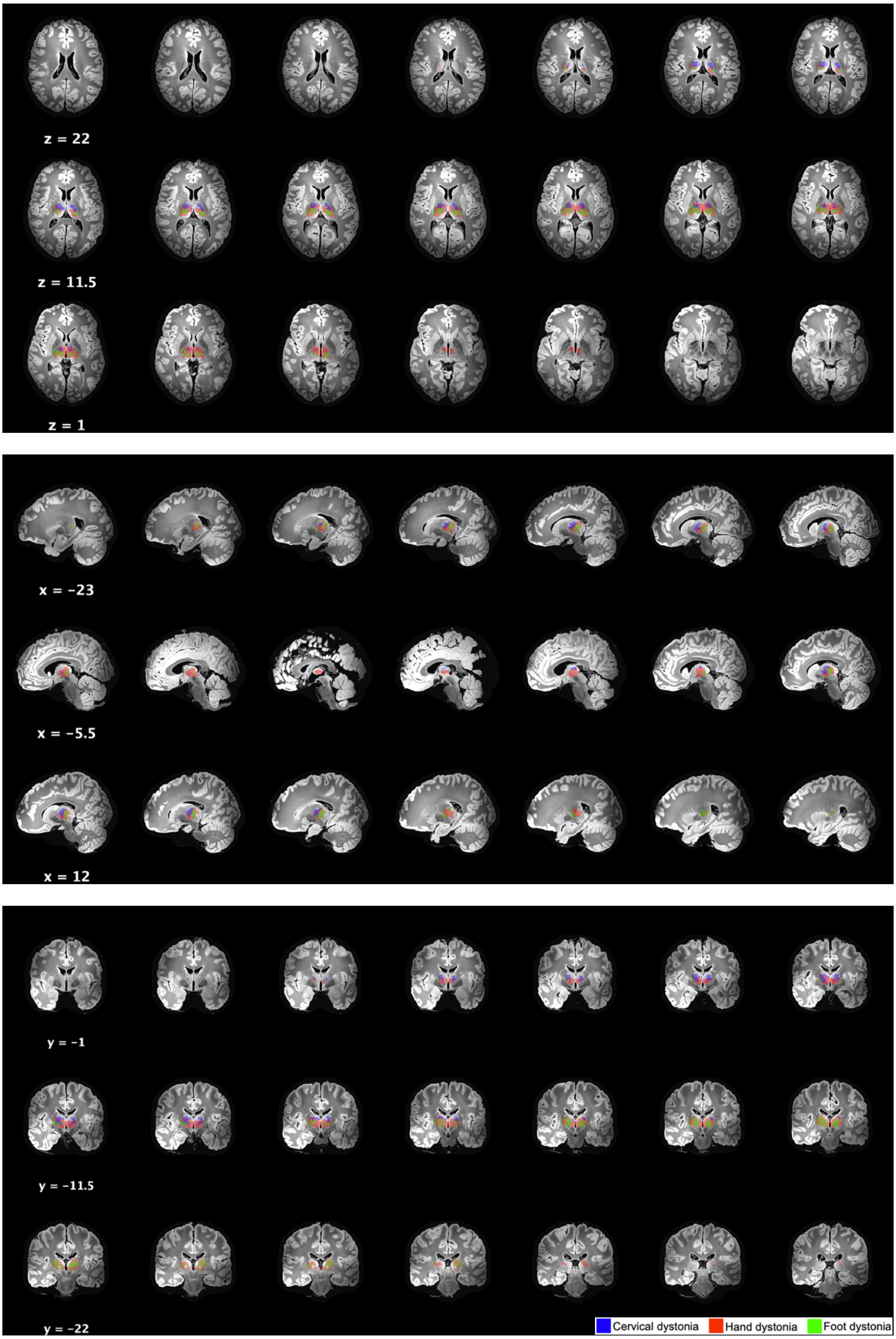

Supplementary File 13B. Additional slices of cervical, hand, and foot dystonia lesion networks in the thalamus.

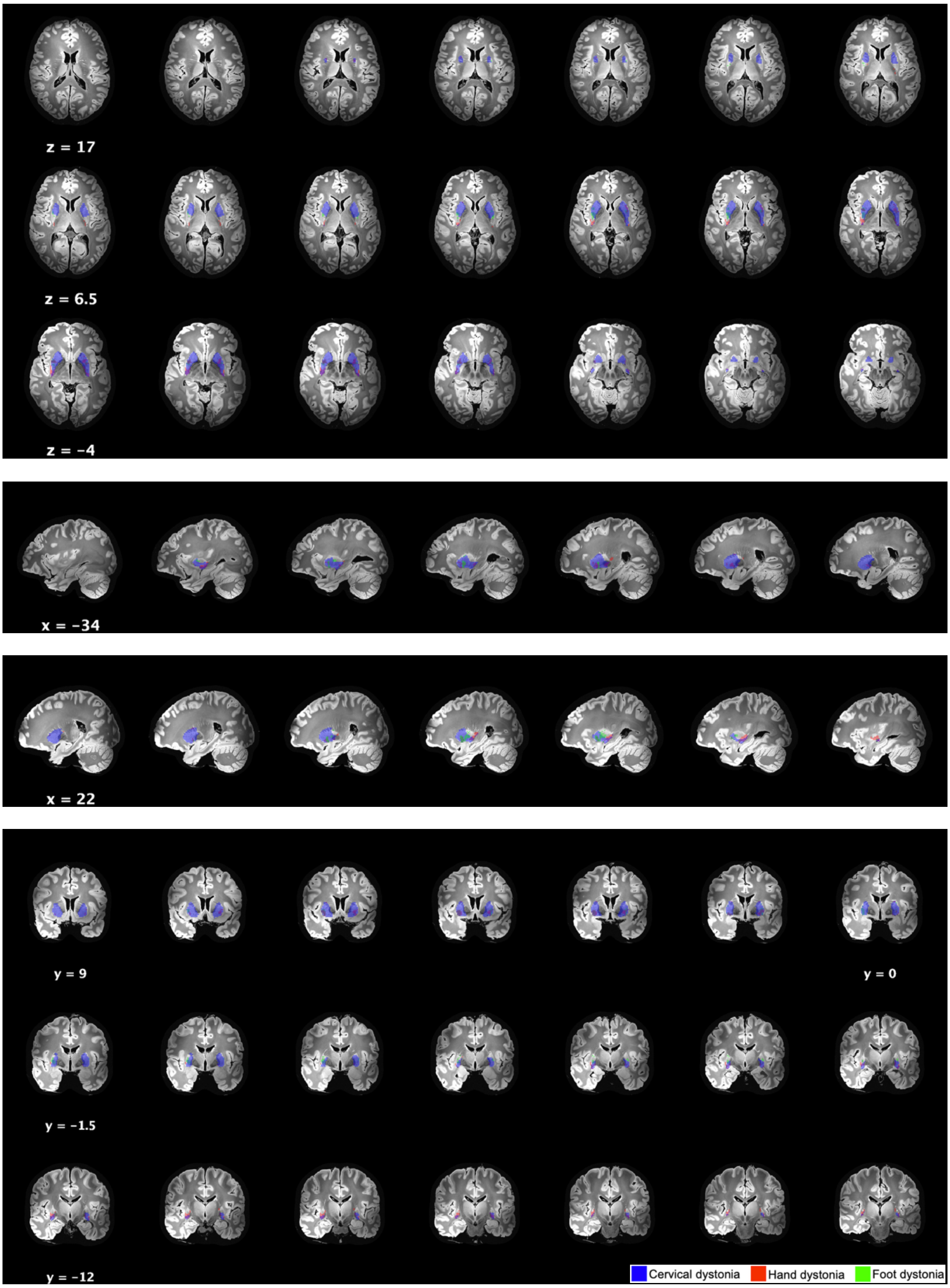

Supplementary File 13C. Additional slices of cervical, hand, and foot dystonia lesion networks in the putamen.
